# Prevalence, characteristics, and determinants of chest pain among physicians in the Middle East and North Africa: A multinational cross-sectional study

**DOI:** 10.1371/journal.pone.0355946

**Published:** 2026-08-31

**Authors:** Saadi M. Saleh, Weam Aldiban, Alaa Aljamala, Khaled Gadallah, Omar Habib, Mansour Algazar, Omar Abdalazeez, Safia Abdi, Noorulhuda Oudah, Hamza I. Alhamdan, Bushra M. Rageh, DARS Consortium, Rasha Shaker Eldesouky

**Affiliations:** 1 Department of Internal Medicine, Hamad Medical Corporation, Doha, Qatar; 2 Faculty of Medicine, Benha University, Benha, Egypt; 3 Faculty of Pharmacy, International University for Science and Technology (IUST), Daraa, Syrian Arab Republic; 4 Faculty of Medicine, Menoufia University, Menoufia, Egypt; 5 Faculty of Medicine, Portsaid University, Portsaid, Egypt; 6 Faculty of Medicine, Alazhar University, Asyut, Egypt; 7 Faculty of Medicine, Alazhar University, Cairo, Egypt; 8 Omdurman Islamic University Faculty of Medicine, Khartoum, Sudan; 9 Alkindy College of Medicine, Baghdad University, Baghdad, Iraq; 10 Faculty of Medicine, Hashemite University, Zarqa, Jordan; 11 Department of Physiology, Faculty of Medicine, Sana’a University, Sana’a, Yemen; 12 Vice Dean of Education and Student Affairs, Faculty of Medicine, Benha University, Banha, Egypt; Mount Sinai Hospital, UNITED STATES OF AMERICA

## Abstract

**Background:**

Physicians face substantial occupational, psychological, and cardiovascular risk factors predisposing them to chest pain, yet no large-scale study has examined this in the Middle East and North Africa (MENA). This study determined the prevalence, characteristics, and determinants of chest pain among MENA physicians.

**Methods:**

This multinational cross-sectional study enrolled 6,224 physicians from 12 MENA countries (August–December 2023). Chest pain was classified using the Rose Angina Questionnaire. Anxiety and panic disorder were assessed using the Generalized Anxiety Disorder-7 (GAD-7) and Panic Disorder Screener (PADIS). Binary and multinomial logistic regression identified independent determinants.

**Results:**

Overall, 795 (12.8%) met criteria for definitive anginal pain and 2,836 (45.6%) for possible anginal pain. In the adjusted binary model, panic disorder was the strongest predictor (AOR = 2.76, 95% CI: 2.44–3.13), followed by physical comorbidities (AOR = 1.91), sleep deprivation (AOR = 1.61), female sex (AOR = 1.49), anxiety disorder (AOR = 1.38), and obesity (AOR = 1.30). All nicotine product categories were independently associated with chest pain. Multinomial analysis revealed stronger associations of panic disorder (AOR = 3.52) and tobacco use with definitive anginal pain, while electronic nicotine delivery system (ENDS) use was significant only for possible anginal pain. Middle-income country physicians had higher odds than those from high-income countries (AOR = 1.71).

**Conclusion:**

Chest pain was highly prevalent among MENA physicians, and was strongly associated with panic disorder, anxiety, sleep deprivation, and nicotine product use. Integrated occupational health programmes addressing both psychological and cardiovascular risk are urgently needed.

## Introduction

Chest pain is among the most clinically significant and diagnostically complex symptoms encountered in medical practice. It accounts for approximately 6–8% of all emergency department visits globally and represents a leading cause of unscheduled healthcare utilization [1,2]. While chest pain carries an important association with serious cardiovascular conditions — including acute coronary syndromes, stable angina pectoris, and aortic disease — the majority of chest pain presentations in community and outpatient settings are non-cardiac in origin, arising from gastrointestinal, musculoskeletal, pulmonary, or psychological causes [3–5]. This diagnostic complexity is compounded by the substantial overlap between cardiac and non-cardiac presentations, particularly in the context of anxiety disorders and panic disorder, which can produce chest pain that closely mimics classical angina both clinically and experientially [6,7].

The Middle East and North Africa (MENA) region presents a distinctive epidemiological context for cardiovascular disease. Cardiovascular events in this region occur at younger ages than in Western populations, driven by a rapidly increasing prevalence of cardiovascular risk factors including obesity, type 2 diabetes, dyslipidaemia, and tobacco use [8,9]. Despite this growing burden, population-level data on chest pain symptoms — as distinct from established cardiovascular disease endpoints — remain sparse, and the determinants of chest pain in this region are poorly characterised.

Healthcare workers constitute one of the most occupationally stressed professional groups globally. The demands of clinical practice — encompassing prolonged working hours, shift work, night duties, emotional labour, exposure to patient suffering and death, and the perpetual pressure of clinical decision-making — impose a substantial and sustained physiological burden on those who deliver care, one that is increasingly reflected in elevated rates of cardiovascular symptoms including chest pain [10,11]. Growing attention has also been directed toward broader downstream physical health consequences of occupational stress in this group, including musculoskeletal disorders, metabolic dysfunction, and cardiovascular disease [10,12].

Healthcare workers in the MENA region face a dual vulnerability: they are exposed to the same population-level cardiovascular risk factors as the general public, while simultaneously bearing the disproportionate physiological consequences of demanding occupational environments. Furthermore, psychological comorbidities — including anxiety disorders, panic disorder, and depression — are prevalent in this workforce and are increasingly recognised as independent contributors to both cardiac and non-cardiac chest pain through mechanisms including chronic autonomic dysregulation, systemic inflammation, and heightened somatic symptom perception [7,13,14]. Moreover, the region’s healthcare systems span a wide range of socioeconomic development levels, from high-income Gulf states to lower-middle-income countries, creating substantial heterogeneity in working conditions, staffing ratios, and access to occupational health support — all of which may meaningfully influence chest pain risk [8,15].

Despite this convergence of occupational, psychological, and cardiovascular risk factors, no large-scale study has examined the prevalence and determinants of chest pain specifically among healthcare workers in the MENA region. Existing studies from the region have focused predominantly on cardiovascular disease endpoints in the general population [8,9], while the occupational health literature on MENA healthcare workers has centred on burnout, mental health, and infection-related exposures [13,16,17]. Therefore, this study aimed primarily to estimate the prevalence of chest pain and, secondarily, to describe its characteristics and identify its determinants among physicians in the MENA region.

## Methods

### Study design, setting, and participants

This is a multinational cross-sectional study conducted among physicians from 12 MENA countries between August 25 and December 25, 2023 (S1 Table). Participants were eligible if they were physicians actively practising medicine within the MENA region and aged between 24 and 60 years. Only respondents who met both eligibility criteria were included in the analysis. The study was reported in accordance with the Strengthening the Reporting of Observational Studies in Epidemiology (STROBE) checklist (S2 Table) [18].

### Ethical considerations and approval

The study was conducted in accordance with the principles of the Declaration of Helsinki [19], and received ethical approval from the Research Ethics Committee at the Faculty of Medicine, Benha University (REC-FOMBU), Egypt (registration number: RC.12.8.2023), and from Alrazi University, Sana’a City, Yemen (reference number: 016/FMHS/2023). Each participant was provided with a cover page detailing the study’s purpose and containing statements regarding confidentiality, anonymity, voluntary participation, and data privacy. Completion of the questionnaire was contingent on providing electronic informed consent, and participants retained the right to withdraw at any stage without consequence.

### Data collection tools and procedures

Data were collected using an English-language self-administered online questionnaire developed via Google Forms (Google LLC). The survey was disseminated through a network of collaborators across participating countries via social media platforms, including Facebook, Telegram, WhatsApp, Twitter, and Snapchat. Collaborators facilitated distribution within relevant academic and professional communities, and participants were screened for eligibility prior to receiving access to the survey link. To ensure data integrity, the survey was configured to permit only one response per participant. Responses were additionally reviewed manually, and those deemed invalid — including entries with repetitive or identical answer patterns — were excluded from the final analysis.

The survey comprised four sections. The first section collected sociodemographic information (S1 File), including age, sex, marital status, country of residence, and nationality, alongside personal history (nicotine product use, alcohol consumption, recreational drug use, and caffeinated drink consumption). Occupational variables included seniority rank, medical specialty, place of work, years of experience, and working hours per week. Health-related variables included self-reported height and weight for body mass index (BMI) calculation, subjective sleep deprivation, and self-reported physical and psychiatric comorbidities. The country income level variable was created by grouping the participating countries into high, middle, and low income according to the World Bank 2023 income classification [20,21].

The second section contained the Rose Angina Questionnaire (RAQ), a validated epidemiological instrument for classifying chest pain based on standardised symptom criteria [22]. First developed in 1962, the RAQ has been used extensively in multinational epidemiological studies and translated into nine languages [23]. It demonstrates high specificity (80–95%) with variable sensitivity (19–83%) depending on the reference standard applied [23,24]. The RAQ includes structured questions assessing the presence, location, character, and triggers of chest pain, and its relationship to physical exertion and rest. Chest pain was classified into three categories: no chest pain, possible anginal pain, and definitive anginal pain. Participants were classified as having no chest pain if they reported never experiencing chest pain or discomfort. Definitive anginal pain was defined as chest pain precipitated by exertion (walking uphill, hurrying, or walking at an ordinary pace), causing the participant to stop or slow down, relieved by rest within 10 minutes, and localised either to the sternum (any level) or the left arm and left anterior chest. Participants reporting chest pain that did not fulfil all these criteria were classified as having possible anginal pain.

The third section assessed anxiety symptoms using the Generalized Anxiety Disorder-7 (GAD-7) questionnaire, a validated seven-item self-administered screening tool. Each item is rated on a four-point Likert scale from 0 (“not at all”) to 3 (“nearly every day”), yielding a total score of 0–21. A cut-off score of ≥10 was used to screen for participants with clinically significant anxiety symptoms, consistent with validation studies reporting sensitivity of 89% and specificity of 82% at this threshold [25]. The term “anxiety disorder” is used thereafter for brevity to denote screen-positive status rather than confirmed diagnoses.

The fourth section evaluated panic disorder using the Panic Disorder Screener (PADIS), a brief validated instrument designed for community-based screening. The PADIS comprises four items: an initial screening question assessing the frequency of panic attacks in the past month (scored 0–4 on a five-point Likert scale), followed by three items evaluating associated symptoms and functional impact (each scored 0–3), yielding a total score of 0–13. Participants who reported no panic attacks on the initial item were not required to complete the remaining items, and their scores were recorded as zero. A cut-off score of ≥4 was used to screen for participants with clinically significant panic disorder symptoms, corresponding to a sensitivity of 77% and specificity of 84% [26]. The term “panic disorder” is used thereafter for brevity to denote screen-positive status rather than confirmed diagnoses.

### Sample size and sampling

The minimum required sample size was calculated a priori using the Scalex SP calculator [27], based on an expected prevalence of chest pain of 3.5%, as reported by Wang et al. [28]. A minimum of 424 participants was required to estimate this prevalence with an absolute precision of ±1.75% and 95% confidence, yielding an anticipated confidence interval of 1.75% to 5.25%. Participants were recruited using convenience sampling. The final analytic sample of 6,224 participants substantially exceeded the minimum required size, conferring greater statistical precision and power than originally planned, and providing sufficient power for the multivariable binary and multinomial logistic regression analyses across 12 countries and multiple covariates [29].

### Statistical analysis

All statistical analyses were performed using jamovi (version 2.3; The jamovi Project, Sydney, Australia). The survey was configured with mandatory fields, so no missing data occurred. Continuous variables were summarised as means with standard deviations (SD), and categorical variables as frequencies and percentages. Between-group differences across the three categories of the chest-pain outcome — definitive anginal pain, possible anginal pain, and no chest pain — were examined using one-way analysis of variance (ANOVA) for continuous variables and Pearson’s chi-squared test for categorical variables.

To identify factors independently associated with chest pain, a binary logistic regression analysis was conducted with chest pain (combining definitive and possible anginal pain) as the dependent variable, and no chest pain as the reference category. Crude and adjusted odds ratios (OR and AOR) with corresponding 95% confidence intervals (CI) and *p*-values were reported. Covariates were selected for inclusion in the initial adjusted model based on a combination of clinical relevance and statistical significance in univariate analysis (*p* < 0.20); those that were non-significant and did not improve model fit were removed from the final model. The adjusted binary model included the following variables: age, sex, body mass index category, physical comorbidities, subjective sleep deprivation, anxiety disorder, panic disorder, nicotine product use, seniority rank, and country income level. Multicollinearity among independent variables was assessed using variance inflation factors (VIF), with all values indicating no significant collinearity (VIF < 5). Model performance for the binary logistic regression model was evaluated using Akaike Information Criterion (AIC), McFadden’s pseudo-R², and area under the receiver operating characteristic curve (AUC), along with sensitivity, specificity, and overall classification accuracy.

A sensitivity analysis was conducted by additionally including psychiatric comorbidities in the adjusted binary model to assess the robustness of the primary estimates against residual confounding from overlapping psychological variables.

To examine whether the determinants of chest pain differed between subtypes, multinomial logistic regression analysis was performed with chest pain as a three-level outcome — definitive anginal pain, possible anginal pain, and no chest pain — using the no-chest-pain group as the reference category. This approach allowed simultaneous estimation of subtype-specific associations, providing greater epidemiological granularity than binary modelling alone. Adjusted odds ratios with 95% CIs and *p*-values were reported for each outcome category. Variable selection followed the same criteria as the binary model, except that seniority rank was not retained in the adjusted multinomial model as it did not reach statistical significance for either chest pain outcome category. Model fit was assessed using AIC and McFadden’s pseudo-R².

Medical specialty was evaluated as a candidate covariate but excluded from the final models because of instability caused by sparse cells across 23 specialty categories.

To enhance transparency and reproducibility, complete univariate results for all candidate variables — including those not retained in the adjusted models — are presented in S3 and S4 Tables for the binary and multinomial analyses, respectively. All reported *p*-values are two-tailed. Odds ratios and confidence interval bounds are presented rounded to two decimal places, consistent with standard epidemiological reporting practice. No adjustments were made for multiple comparisons, as the analyses were hypothesis-driven and based on pre-specified clinically relevant variables rather than exploratory data mining.

## Results

### Sample characteristics

A total of 6,224 physicians were included in the analysis. Overall, 3,631 (58.3%) reported having experienced chest pain, while 2,593 (41.7%) had never experienced chest pain. Among those reporting chest pain, 795 (12.8% of the total sample) met criteria for definitive anginal pain and 2,836 (45.6%) for possible anginal pain. The characteristics of the study participants are presented in Table 1.

**Table 1:** Sample characteristics.

Variables		Experienced definitive anginal pain (N = 795)	Experienced possible anginal pain (N = 2836)	Never experienced chest pain (N = 2593)	Total (N = 6224)	p value
Age, mean (SD)		29.3 (6.0)	29.1 (5.9)	29.1 (5.9)	29.1 (5.9)	0.797^1^
Sex (male)		352.0 (44.3%)	1533.0 (54.1%)	1564.0 (60.3%)	3449.0 (55.4%)	< 0.001^2^
Marital status	Single	493.0 (62.0%)	1845.0 (65.1%)	1726.0 (66.6%)	4064.0 (65.3%)	0.081^2^
	Married	288.0 (36.2%)	932.0 (32.9%)	833.0 (32.1%)	2053.0 (33.0%)	
	Separated	4.0 (0.5%)	23.0 (0.8%)	9.0 (0.3%)	36.0 (0.6%)	
	Divorced	10.0 (1.3%)	29.0 (1.0%)	20.0 (0.8%)	59.0 (0.9%)	
	Widowed	0.0 (0.0%)	7.0 (0.2%)	5.0 (0.2%)	12.0 (0.2%)	
Body mass index (kg/m^2^), mean (SD)		25.9 (4.9)	25.9 (5.2)	25.5 (4.9)	25.7 (5.0)	0.016^1^
Body mass index categories	Healthy weight (18.5–24.9 kg/m²)	329.0 (41.4%)	1233.0 (43.5%)	1214.0 (46.8%)	2776.0 (44.6%)	< 0.001^2^
	Underweight (<18.5 kg/m²)	32.0 (4.0%)	100.0 (3.5%)	83.0 (3.2%)	215.0 (3.5%)	
	Overweight (25.0–29.9 kg/m²)	307.0 (38.6%)	1005.0 (35.4%)	947.0 (36.5%)	2259.0 (36.3%)	
	Obesity (≥30.0 kg/m²)	127.0 (16.0%)	498.0 (17.6%)	349.0 (13.5%)	974.0 (15.6%)	
Exercise frequency	Non-exercising	458.0 (57.6%)	1507.0 (53.1%)	1393.0 (53.7%)	3358.0 (54.0%)	0.010^2^
	1-2 times/week	190.0 (23.9%)	703.0 (24.8%)	588.0 (22.7%)	1481.0 (23.8%)	
	3-5 times/week	119.0 (15.0%)	548.0 (19.3%)	538.0 (20.7%)	1205.0 (19.4%)	
	>5 times/week	28.0 (3.5%)	78.0 (2.8%)	74.0 (2.9%)	180.0 (2.9%)	
Physical comorbidities (yes)		102.0 (12.8%)	430.0 (15.2%)	196.0 (7.6%)	728.0 (11.7%)	< 0.001^2^
Psychiatric comorbidities (yes)		35.0 (4.4%)	158.0 (5.6%)	61.0 (2.4%)	254.0 (4.1%)	< 0.001^2^
Subjective sleep deprivation (yes)		440.0 (55.3%)	1417.0 (50.0%)	856.0 (33.0%)	2713.0 (43.6%)	< 0.001^2^
Anxiety disorder (yes)		372.0 (46.8%)	1218.0 (42.9%)	682.0 (26.3%)	2272.0 (36.5%)	< 0.001^2^
Panic disorder (yes)		424.0 (53.3%)	1252.0 (44.1%)	536.0 (20.7%)	2212.0 (35.5%)	< 0.001^2^
Nicotine product use	non-use	650.0 (81.8%)	2280.0 (80.4%)	2200.0 (84.8%)	5130.0 (82.4%)	< 0.001^2^
	Exclusive ENDS use	44.0 (5.5%)	184.0 (6.5%)	133.0 (5.1%)	361.0 (5.8%)	
	Exclusive tobacco use	83.0 (10.4%)	271.0 (9.6%)	206.0 (7.9%)	560.0 (9.0%)	
	Dual use	18.0 (2.3%)	101.0 (3.6%)	54.0 (2.1%)	173.0 (2.8%)	
Caffeinated drink consumption (yes)		724.0 (91.1%)	2548.0 (89.8%)	2330.0 (89.9%)	5602.0 (90.0%)	0.564^2^
Alcohol consumption (yes)		26.0 (3.3%)	109.0 (3.8%)	72.0 (2.8%)	207.0 (3.3%)	0.091^2^
Recreational drug use (yes)		35.0 (4.4%)	117.0 (4.1%)	86.0 (3.3%)	238.0 (3.8%)	0.198^2^
Academic/professional degree	Bachelor’s degree	613.0 (77.1%)	2246.0 (79.2%)	2081.0 (80.3%)	4940.0 (79.4%)	0.179^2^
	Diploma	23.0 (2.9%)	69.0 (2.4%)	66.0 (2.5%)	158.0 (2.5%)	
	Fellowship or board	47.0 (5.9%)	201.0 (7.1%)	150.0 (5.8%)	398.0 (6.4%)	
	Master’s degree	79.0 (9.9%)	219.0 (7.7%)	217.0 (8.4%)	515.0 (8.3%)	
	Doctorate degree	33.0 (4.2%)	101.0 (3.6%)	79.0 (3.0%)	213.0 (3.4%)	
Seniority	House officer	208.0 (26.2%)	678.0 (23.9%)	733.0 (28.3%)	1619.0 (26.0%)	0.007^2^
	General practitioner	230.0 (28.9%)	865.0 (30.5%)	770.0 (29.7%)	1865.0 (30.0%)	
	Resident	238.0 (29.9%)	923.0 (32.5%)	765.0 (29.5%)	1926.0 (30.9%)	
	Attending	119.0 (15.0%)	370.0 (13.0%)	325.0 (12.5%)	814.0 (13.1%)	
Years of experience, years		3.5 (5.0)	3.4 (4.9)	3.2 (4.6)	3.3 (4.8)	0.069^1^
Working hours/week, mean (SD)		43.7 (25.2)	44.7 (26.2)	42.3 (24.5)	43.6 (25.4)	0.003^1^
Working hours/week categories	<40 hours/week	339.0 (42.6%)	1170.0 (41.3%)	1118.0 (43.1%)	2627.0 (42.2%)	0.170^2^
	40-80 hours/week	401.0 (50.4%)	1448.0 (51.1%)	1318.0 (50.8%)	3167.0 (50.9%)	
	>80 hours/week	55.0 (6.9%)	218.0 (7.7%)	157.0 (6.1%)	430.0 (6.9%)	
Country income	Low	186.0 (23.4%)	668.0 (23.6%)	751.0 (29.0%)	1605.0 (25.8%)	< 0.001^2^
	Middle	532.0 (66.9%)	1957.0 (69.0%)	1531.0 (59.0%)	4020.0 (64.6%)	
	High	77.0 (9.7%)	211.0 (7.4%)	311.0 (12.0%)	599.0 (9.6%)	

Abbreviations: ENDS, electronic nicotine delivery system.

^1^Linear Model ANOVA.

^2^Pearson’s Chi-squared test.

The mean age of participants was 29.1 years (SD = 5.9), with no statistically significant difference across the three groups (*p* = 0.797). The majority were males (55.4%), singles (65.3%), and held a bachelor’s degree (79.4%). The mean BMI was 25.7 kg/m² (SD = 5.0), with a statistically significant difference across groups (*p* = 0.016). Obesity was more prevalent among those with possible anginal pain (17.6%) compared to those who never experienced chest pain (13.5%) (*p* < 0.001).

Regarding psychological and sleep-related factors, subjective sleep deprivation was reported by 55.3% of participants with definitive anginal pain, 50.0% with possible anginal pain, and 33.0% of those who never experienced chest pain (*p* < 0.001). Anxiety disorder was present in 46.8%, 42.9%, and 26.3% of the three groups respectively, and panic disorder in 53.3%, 44.1%, and 20.7% (both *p* < 0.001). Physical comorbidities were reported by 12.8%, 15.2%, and 7.6% across groups (*p* < 0.001), and psychiatric comorbidities by 4.4%, 5.6%, and 2.4% (*p* < 0.001).

Regarding occupational characteristics, seniority rank differed significantly across groups (*p* = 0.007). The mean working hours per week were 43.7 (SD = 25.2) for the definitive anginal pain group, 44.7 (SD = 26.2) for possible anginal pain, and 42.3 (SD = 24.5) for those with no chest pain (*p* = 0.003). Country income level also differed significantly across groups (*p* < 0.001), with a higher proportion of participants from high-income countries in the never-experienced-chest-pain group (12.0%) compared to the definitive anginal pain group (9.7%).

**Fig 1:**
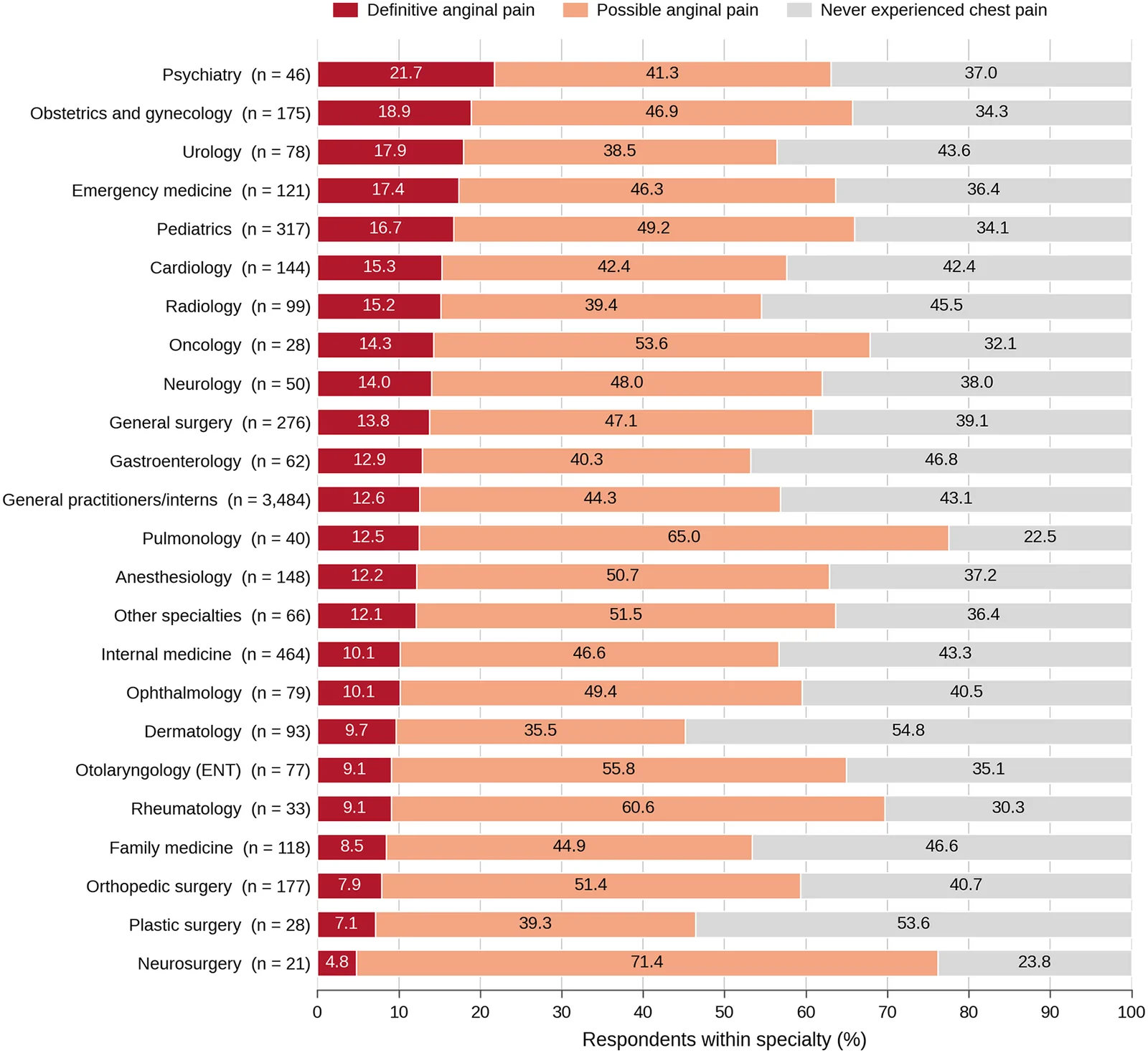
Chest pain distribution by medical specialty. Stacked bars show the percentage of physicians within each specialty classified by the Rose Angina Questionnaire as having definitive anginal pain, possible anginal pain, or no chest pain. Specialties are ordered by descending prevalence of definitive anginal pain, with respondent numbers in parentheses. Distribution differed significantly across specialties (χ² (46) = 74.9, p = 0.005; N = 6,224).

Chest pain distribution varied significantly across medical specialties (*p* = 0.005) (Fig 1). Psychiatrists reported the highest prevalence of definitive anginal pain (21.7%), followed by Obstetrics & Gynecology (18.9%), Urology (17.9%), and Emergency Medicine (17.4%), while Neurosurgery had the lowest (4.8%).

### Chest pain characteristics

The characteristics of chest pain among the 3,631 participants who experienced chest pain (definitive or possible anginal pain) are presented in Table 2. The most common pain sites in the definitive anginal pain group were the sternum upper middle (65.2%) and sternum lower (42.3%), whereas in the possible anginal pain group, the left anterior chest was the most commonly reported site (53.0%), followed by the sternum upper middle (32.2%). Chest pain had occurred more than three times in 54.3% and 55.3% of the definitive and possible anginal pain groups, respectively.

**Table 2:** Chest pain characteristics.

Variables		Experienced definitive anginal pain (N = 795)	Experienced possible anginal pain (N = 2836)	Total (N = 3631)
Site	Left anterior chest	166.0 (20.9%)	1503.0 (53.0%)	1669.0 (46.0%)
	Left arm	61.0 (7.7%)	198.0 (7.0%)	259.0 (7.1%)
	Right chest	1.0 (0.1%)	56.0 (2.0%)	57.0 (1.6%)
	Sternum (lower)	336.0 (42.3%)	576.0 (20.3%)	912.0 (25.1%)
	Sternum (upper middle)	518.0 (65.2%)	912.0 (32.2%)	1430.0 (39.4%)
	All over chest	0.0 (0.0%)	49.0 (1.7%)	49.0 (1.3%)
	Others	3.0 (0.4%)	37.0 (1.3%)	40.0 (1.1%)
Experienced chest pain/discomfort >3 times		432.0 (54.3%)	1569.0 (55.3%)	2001.0 (55.1%)
Factors that induced chest pain/discomfort	Excitement or emotion	509.0 (64.0%)	1700.0 (59.9%)	2209.0 (60.8%)
	Stooping	46.0 (5.8%)	132.0 (4.7%)	178.0 (4.9%)
	Eating	92.0 (11.6%)	346.0 (12.2%)	438.0 (12.1%)
	Breathing	176.0 (22.1%)	513.0 (18.1%)	689.0 (19.0%)
	Cold wind	186.0 (23.4%)	440.0 (15.5%)	626.0 (17.2%)
	Coughing	273.0 (34.3%)	624.0 (22.0%)	897.0 (24.7%)
Chest pain gets worse with a chest cold or a bad cough		432.0 (54.3%)	1086.0 (38.3%)	1518.0 (41.8%)
General description of chest pain/discomfort	Pain	245.0 (30.8%)	1034.0 (36.5%)	1279.0 (35.2%)
	Discomfort	546.0 (68.7%)	1767.0 (62.3%)	2313.0 (63.7%)
	Both	2.0 (0.3%)	7.0 (0.2%)	9.0 (0.2%)
	Can’t specify	2.0 (0.3%)	28.0 (1.0%)	30.0 (0.8%)
Specific description of the chest pain/discomfort	Burning	109.0 (13.7%)	359.0 (12.7%)	468.0 (12.9%)
	Pressure	286.0 (36.0%)	765.0 (27.0%)	1051.0 (28.9%)
	Stabbing	193.0 (24.3%)	876.0 (30.9%)	1069.0 (29.4%)
	Tightness	349.0 (43.9%)	1080.0 (38.1%)	1429.0 (39.4%)
	Heaviness	372.0 (46.8%)	878.0 (31.0%)	1250.0 (34.4%)

Emotion/excitement was the most frequently reported trigger in both groups (64.0% definitive; 59.9% possible), followed by coughing (34.3% vs. 22.0%) and cold wind (23.4% vs. 15.5%). Worsening with a chest cold or bad cough was reported by 54.3% of those with definitive anginal pain compared to 38.3% of those with possible anginal pain. In terms of pain quality, tightness and heaviness were more prevalent in the definitive anginal pain group, while stabbing pain was more common in the possible anginal pain group. The majority of participants described their experience as discomfort rather than pain across both groups (68.7% and 62.3%, respectively).

### Factors associated with chest pain: Binary logistic regression

The crude and adjusted binary logistic regression analyses examining factors associated with combined chest pain (definitive and possible anginal pain vs. never experienced chest pain) are presented in Table 3. In the adjusted model, female sex was significantly associated with higher odds of chest pain (AOR = 1.49, 95% CI: 1.33–1.68, *p* < 0.001). Among BMI categories, obesity was the only category significantly associated with chest pain after adjustment (AOR = 1.30, 95% CI: 1.10–1.54, *p* = 0.002). Age was not significantly associated with chest pain in either the crude or adjusted analysis.

**Table 3:** Factors associated with chest pain (combined possible and definite anginal chest pain): Crude and adjusted analysis.

Variable	OR (95% CI)	p-value	AOR* (95% CI)	p-value
Age (years)	1.00 (0.99–1.01)	0.71	0.99 (0.98–1.00)	0.11
Sex (reference: male)	1.41 (1.27–1.56)	<0.001	1.49 (1.33–1.68)	<0.001
Body mass index (reference: healthy weight)				
Underweight	1.24 (0.93–1.64)	0.144	1.31 (0.97–1.77)	0.084
Overweight	1.08 (0.96–1.20)	0.197	1.06 (0.93–1.20)	0.39
Obese	1.39 (1.20–1.62)	<0.001	1.30 (1.10–1.54)	0.002
Physical comorbidities (reference: no)	2.10 (1.77–2.49)	<0.001	1.91 (1.59–2.30)	<0.001
Sleep deprivation (reference: no)	2.12 (1.91–2.36)	<0.001	1.61 (1.43–1.80)	<0.001
Anxiety disorder (reference: no)	2.18 (1.96–2.43)	<0.001	1.38 (1.23–1.56)	<0.001
Panic disorder (reference: no)	3.29 (2.93–3.69)	<0.001	2.76 (2.44–3.13)	<0.001
Nicotine product use (reference: non-users)				
Exclusive ENDS users	1.29 (1.03–1.61)	0.025	1.33 (1.05–1.69)	0.018
Exclusive tobacco users	1.29 (1.08–1.55)	0.006	1.29 (1.06–1.57)	0.013
Dual users	1.65 (1.19–2.29)	0.002	1.53 (1.08–2.16)	0.016
Seniority (reference: house officer)				
General practitioner	1.18 (1.03–1.35)	0.018	1.13 (0.98–1.31)	0.104
Resident	1.26 (1.10–1.44)	<0.001	1.18 (1.01–1.37)	0.042
Attending	1.24 (1.05–1.48)	0.012	1.34 (1.03–1.76)	0.032
Country income level (reference: high)				
Low	1.23 (1.02–1.48)	0.032	1.23 (1.00–1.51)	0.05
Middle	1.76 (1.48–2.09)	<0.001	1.71 (1.42–2.07)	<0.001

Abbreviations: OR, odds ratio; AOR, adjusted odds ratio; 95% CI, 95% confidence interval; ENDS, electronic nicotine delivery system.

The reference is the never-experienced chest pain group.

*Model fit: AIC = 7,704; McFadden’s R² = 0.0930; AUC = 0.704; Sensitivity = 0.754; Specificity = 0.547; Accuracy = 0.667 (the cut-off value is set to 0.5).

Physical comorbidities were significantly associated with increased odds of chest pain (AOR = 1.91, 95% CI: 1.59–2.30, *p* < 0.001). Among psychological factors, panic disorder showed the strongest association (AOR = 2.76, 95% CI: 2.44–3.13, *p* < 0.001), followed by sleep deprivation (AOR = 1.61, 95% CI: 1.43–1.80, *p* < 0.001) and anxiety disorder (AOR = 1.38, 95% CI: 1.23–1.56, *p* < 0.001).

All three nicotine product use categories were independently associated with chest pain after adjustment. Dual users had the highest odds (AOR = 1.53, 95% CI: 1.08–2.16, *p* = 0.016), followed by exclusive electronic nicotine delivery system (ENDS) users (AOR = 1.33, 95% CI: 1.05–1.69, *p* = 0.018) and exclusive tobacco users (AOR = 1.29, 95% CI: 1.06–1.57, *p* = 0.013).

Regarding seniority rank, residents (AOR = 1.18, 95% CI: 1.01–1.37, *p* = 0.042) and attending physicians (AOR = 1.34, 95% CI: 1.03–1.76, *p* = 0.032) had significantly higher odds of chest pain compared to house officers. Country income level was also a significant predictor, with middle-income country participants showing higher odds of chest pain compared to high-income country participants (AOR = 1.71, 95% CI: 1.42–2.07, *p* < 0.001). Participants from low-income countries showed borderline significance (AOR = 1.23, 95% CI: 1.00–1.51, *p* = 0.050).

In a sensitivity analysis additionally including psychiatric comorbidities in the adjusted binary model, all primary predictors remained materially unchanged, with adjusted odds ratios within ±3% of the main model and preserved direction and statistical significance; psychiatric comorbidity was itself an independent predictor (AOR = 1.49, 95% CI: 1.09–2.04, *p* = 0.012).

### Factors associated with chest pain: Multinomial logistic regression

To distinguish factors associated with definitive anginal pain from those associated with possible anginal pain, a multinomial logistic regression analysis was performed with never-experienced chest pain as the reference category. Results are presented in Table 4.

**Table 4:** Multinomial logistic regression analysis of factors associated with chest pain.

Variable	Definitive anginal pain				Possible anginal pain			
	OR (95% CI)	p-value	AOR* (95% CI)	p-value	OR (95% CI)	p-value	AOR* (95% CI)	p-value
Age	1.01 (0.99–1.02)	0.501	1.00 (0.99–1.02)	0.757	1.00 (0.99–1.01)	0.862	1.00 (0.99–1.01)	0.706
Sex (reference: male)	1.91 (1.63–2.25)	<0.001	2.06 (1.72–2.47)	<0.001	1.29 (1.16–1.44)	<0.001	1.36 (1.21–1.54)	<0.001
Body mass index (reference: healthy weight)								
Underweight	1.42 (0.93–2.18)	0.105	1.51 (0.96–2.35)	0.072	1.19 (0.88–1.60)	0.267	1.25 (0.91–1.72)	0.164
Overweight	1.20 (1.00–1.43)	0.048	1.22 (1.01–1.47)	0.041	1.05 (0.93–1.18)	0.469	1.02 (0.90–1.17)	0.725
Obese	1.34 (1.06–1.70)	0.015	1.31 (1.02–1.69)	0.036	1.41 (1.20–1.65)	<0.001	1.31 (1.11–1.56)	0.002
Physical comorbidities (reference: no)	1.80 (1.40–2.32)	<0.001	1.59 (1.22–2.07)	<0.001	2.19 (1.83–2.61)	<0.001	1.99 (1.65–2.40)	<0.001
Sleep deprivation (reference: no)	2.52 (2.14–2.96)	<0.001	1.88 (1.58–2.23)	<0.001	2.03 (1.82–2.26)	<0.001	1.56 (1.38–1.75)	<0.001
Anxiety disorder (reference: no)	2.46 (2.09–2.90)	<0.001	1.38 (1.16–1.66)	<0.001	2.11 (1.88–2.37)	<0.001	1.39 (1.22–1.57)	<0.001
Panic disorder (reference: no)	4.38 (3.70–5.19)	<0.001	3.52 (2.94–4.21)	<0.001	3.03 (2.69–3.42)	<0.001	2.57 (2.26–2.93)	<0.001
Nicotine product use (reference: non-users)								
Exclusive ENDS users	1.12 (0.79–1.59)	0.529	1.30 (0.90–1.88)	0.165	1.34 (1.06–1.68)	0.014	1.35 (1.06–1.73)	0.016
Exclusive tobacco users	1.36 (1.04–1.79)	0.024	1.54 (1.15–2.07)	0.004	1.27 (1.05–1.54)	0.014	1.24 (1.00–1.52)	0.045
Dual users	1.13 (0.66–1.94)	0.661	1.17 (0.67–2.04)	0.591	1.81 (1.29–2.52)	<0.001	1.62 (1.14–2.30)	0.007
Country income level (reference: high)								
Low	1.00 (0.74–1.35)	0.998	1.03 (0.76–1.42)	0.831	1.31 (1.07–1.61)	0.009	1.36 (1.10–1.69)	0.005
Middle	1.40 (1.07–1.84)	0.013	1.46 (1.10–1.94)	0.009	1.88 (1.56–2.27)	<0.001	1.89 (1.55–2.30)	<0.001

Abbreviations: OR, odds ratio; AOR, adjusted odds ratio; 95% CI, 95% confidence interval; ENDS, electronic nicotine delivery system.

The reference is the never-experienced chest pain group.

*Model fit: AIC = 11,487; McFadden’s R² = 0.0688.

Female sex was significantly associated with both definitive anginal pain (AOR = 2.06, 95% CI: 1.72–2.47, *p* < 0.001) and possible anginal pain (AOR = 1.36, 95% CI: 1.21–1.54, *p* < 0.001). Among BMI categories, overweight (AOR = 1.22, 95% CI: 1.01–1.47, *p* = 0.041) and obese (AOR = 1.31, 95% CI: 1.02–1.69, *p* = 0.036) were significantly associated with definitive anginal pain, while only obesity was significant for possible anginal pain (AOR = 1.31, 95% CI: 1.11–1.56, *p* = 0.002). Age was not significantly associated with either outcome.

Physical comorbidities were significantly associated with both definitive anginal pain (AOR = 1.59, 95% CI: 1.22–2.07, *p* < 0.001) and possible anginal pain (AOR = 1.99, 95% CI: 1.65–2.40, *p* < 0.001). Panic disorder demonstrated the strongest association across both outcome categories (definitive: AOR = 3.52, 95% CI: 2.94–4.21; possible: AOR = 2.57, 95% CI: 2.26–2.93; both *p* < 0.001). Sleep deprivation was also significantly associated with both groups (definitive: AOR = 1.88, 95% CI: 1.58–2.23; possible: AOR = 1.56, 95% CI: 1.38–1.75; both *p* < 0.001), as was anxiety disorder (definitive: AOR = 1.38, 95% CI: 1.16–1.66; possible: AOR = 1.39, 95% CI: 1.22–1.57; both *p* < 0.001).

Regarding nicotine product use, exclusive tobacco users were significantly associated with definitive anginal pain (AOR = 1.54, 95% CI: 1.15–2.07, *p* = 0.004), while exclusive ENDS users did not reach significance for definitive anginal pain (AOR = 1.30, 95% CI: 0.90–1.88, *p* = 0.165). For possible anginal pain, all three nicotine use categories were significant: exclusive ENDS users (AOR = 1.35, 95% CI: 1.06–1.73, *p* = 0.016), exclusive tobacco users (AOR = 1.24, 95% CI: 1.00–1.52, *p* = 0.045), and dual users (AOR = 1.62, 95% CI: 1.14–2.30, *p* = 0.007).

Country income level was a significant predictor for possible anginal pain, with participants from middle-income countries having higher odds than those from high-income countries (AOR = 1.89, 95% CI: 1.55–2.30, *p* < 0.001), and participants from low-income countries also showing significantly higher odds (AOR = 1.36, 95% CI: 1.10–1.69, *p* = 0.005). For definitive anginal pain, only middle-income country participants showed a significant association (AOR = 1.46, 95% CI: 1.10–1.94, *p* = 0.009).

## Discussion

This large multinational study examined the prevalence, characteristics, and determinants of chest pain among physicians across 12 MENA countries. Over half reported chest pain experience at least once (58.3%), with 12.8% meeting criteria for definitive anginal pain and 45.6% for possible anginal pain — substantially higher than estimates reported in general MENA region population studies [8,9]. These figures are high for a relatively young working-age professional population and underscore the substantial burden of chest pain symptoms in this understudied occupational group. Collectively, the findings highlight the multifactorial nature of chest pain in this population, with psychological disorders—particularly panic and anxiety disorders—alongside sleep deprivation, physical comorbidities, nicotine product use, obesity, advancing seniority, and country income emerging as key determinants.

The prevalence of chest pain observed in this study substantially exceeds rates reported in general population surveys across the MENA region. National WHO STEPS surveys, which capture self-reported histories of heart attack, angina-type chest pain, and stroke as a composite endpoint, have reported prevalence estimates ranging from approximately 0.9% in Oman [30], to 6.1% in Kuwait [31], with intermediate estimates of 4.4% in Iraq [32], 3.3% in Morocco [33], 5.0% in the occupied Palestinian territory [34], and 4.7% in Lebanon [35]. The considerably higher rates observed in our cohort — 12.8% definitive and 45.6% possible anginal pain — are not directly comparable to these general population estimates for several important methodological reasons. First, the STEPS composite endpoint conflates angina with prior myocardial infarction and stroke. Second, the RAQ, as used in this study, captures a broader spectrum of angina-like symptom experiences — including possible anginal pain — than clinical diagnostic criteria or composite self-report items. Third, the occupational profile of our sample introduces systematic exposure to stressors — prolonged working hours, sleep deprivation, psychological comorbidity — that are independent risk factors for both cardiac and non-cardiac chest pain and are not present at comparable levels in general population cohorts. Taken together, these methodological distinctions suggest that the high chest pain burden observed in this cohort reflects the convergence of an elevated occupational risk environment with the inherent sensitivity of symptom-based classification, rather than indicating a higher prevalence of coronary artery disease per se. Nevertheless, even accounting for these differences, the magnitude of the prevalence observed — particularly for definitive anginal pain — remains clinically significant in a predominantly young professional population and reinforces the case for targeted occupational health surveillance in this group.

The pattern of symptom characteristics supports the validity of the classification approach used in this study. Definitive anginal pain showed a more classical characteristic pattern, with chest pain more frequently described as tightness and heaviness, whereas stabbing pain was more common among those with possible anginal pain. These differences are consistent with the established clinical distinction between more typical anginal pain and atypical or non-cardiac chest pain [36].

Female sex was one of the strongest and most consistent predictors of chest pain in our study, with a more pronounced association for definitive anginal pain than for possible anginal pain. This finding is noteworthy because chest pain, especially obstructive coronary artery disease, is often conceptualized as a predominantly male presentation [37,38]. However, growing evidence suggests that women may experience ischemic symptoms through distinct mechanisms, including coronary microvascular dysfunction, vasospasm, and non-obstructive coronary disease [38–40]. Female healthcare workers may face compounded workplace stressors, including lower autonomy in hierarchical environments, gender-based discrimination, and the simultaneous burden of professional and domestic responsibilities. In the MENA context, these pressures may be further amplified by sociocultural expectations, potentially contributing to the observed sex difference [41,42].

The most significant finding of this study was the strong association between panic disorder and chest pain. Panic disorder emerged as the strongest predictor in the binary model and remained the strongest determinant in both definitive and possible anginal pain categories in the multinomial analysis. This finding is consistent with the established literature identifying panic disorder as a major cause of non-cardiac chest pain [6,43], but the magnitude of the association in this cohort is particularly notable. Chest pain is a frequent feature of panic attacks, and panic disorder is commonly identified among patients presenting with chest pain to emergency and outpatient settings [44,45]. Interestingly, the association with panic disorder was stronger for definitive anginal pain than for possible anginal pain. Although this may seem counterintuitive, it is biologically plausible because panic attacks can produce intense substernal pressure, autonomic symptoms, and exertion-like symptom patterns that closely mimic classical angina [7]. Moreover, panic disorder and cardiovascular disease are not mutually exclusive; chronic autonomic dysregulation, systemic inflammation, and hypothalamic-pituitary-adrenal (HPA) axis activation may increase long-term cardiovascular risk as well [14].

Anxiety disorder was also independently associated with chest pain in both models, with remarkably similar effect sizes across definitive and possible anginal pain. This pattern suggests that anxiety may contribute to chest pain through a broader, non-specific mechanism, likely involving chronic sympathetic activation, somatic hypervigilance, heightened pain perception, and persistent muscle tension [5,46].

Sleep deprivation was another major factor, showing significant associations with chest pain, with a stronger association for definitive anginal pain. This pattern is clinically and biologically plausible. Sleep deprivation is known to increase sympathetic tone, blood pressure, systemic inflammation, endothelial dysfunction, and pain sensitivity. Among healthcare professionals, chronic sleep restriction is common because of shift work, on-call duties, and demanding schedules. The stronger association with definitive anginal pain raises the possibility that sleep disruption may not only intensify symptom perception but may also contribute to genuine cardiovascular stress and ischemia-like symptomatology [47,48].

Physical comorbidities were independently associated with chest pain, with a stronger association for possible anginal pain than for definitive anginal pain. This likely reflects the contribution of non-cardiac conditions such as gastroesophageal reflux disease, respiratory disease, and musculoskeletal disorders, all of which can present with recurrent chest pain [4]. At the same time, some physical comorbidities—particularly hypertension and diabetes—may also contribute to true cardiovascular risk [49]. In a relatively young cohort, however, the overall pattern likely reflects a mixture of both cardiac and non-cardiac mechanisms.

Obesity also emerged as an important determinant. It was the only BMI category that remained significantly associated with chest pain. This is consistent with the multifaceted effects of obesity on chest pain, including increased cardiac workload, gastroesophageal reflux, sleep-disordered breathing, reduced exercise tolerance, and musculoskeletal strain [50]. The near-identical associations across both subtypes suggest that obesity contributes to chest pain through overlapping cardiac and non-cardiac pathways. The additional finding that overweight status was associated with definitive anginal pain but not possible anginal pain may indicate a gradient effect, whereby increasing adiposity is more strongly linked to the more typical anginal phenotype [51].

Nicotine product use showed important subtype-specific patterns. Conventional tobacco use was associated with both definitive and possible anginal pain, with a stronger effect for definitive anginal pain. This is fully consistent with the established cardiotoxic effects of tobacco, including endothelial dysfunction, platelet activation, inflammation, and coronary vasospasm, which can directly contribute to more classical ischemic pain patterns. In contrast, exclusive ENDS use was associated with possible anginal pain but not definitive anginal pain. This distinction is important because it suggests that ENDS-related chest symptoms in this population may be more likely driven by non-cardiac mechanisms such as airway irritation, respiratory inflammation, or nicotine-induced sympathomimetic effects rather than overt myocardial ischemia [52,53]. Dual use was significantly associated with possible anginal pain but not definitive anginal pain, although this may partly reflect limited statistical power because of the relatively small number of dual users. Another possibility is that dual users may have somewhat different cumulative smoke exposure profiles than exclusive tobacco users [53].

Seniority also showed an important association. Residents and attending physicians had higher odds of chest pain than house officers in the adjusted binary model, suggesting a gradient consistent with cumulative occupational stress and allostatic load. More senior physicians often carry greater clinical responsibility, administrative burden, and medicolegal pressure, all of which may amplify stress-related symptom burden [11,54]. Although seniority was not retained in the multinomial model, its significance in the binary model suggests that it may predispose more broadly to chest pain in general rather than to a specific anginal subtype. Working hours, despite showing significance in univariate analyses, did not remain significant in adjusted models, indicating that their effect may be mediated through downstream variables such as sleep deprivation, anxiety, and physical health.

Country income level was another notable determinant. Participants from middle-income countries had higher odds of chest pain relative to those from high-income countries, both in binary and multinomial analyses, while low-income country status was particularly associated with possible anginal pain. These findings likely reflect structural differences in healthcare systems, occupational support, staffing ratios, workload intensity, remuneration, and access to preventive or mental health services [9,15]. The stronger association with possible anginal pain may suggest that psychosocial stressors and untreated non-cardiac conditions contribute substantially to chest pain burden in lower-resource settings [55,56].

### Implications of the findings

Taken together, these findings have several practical implications for occupational health policy and clinical practice. First, the very high prevalence of chest pain supports the need for systematic screening programmes within healthcare institutions that address both cardiovascular and psychological health. Second, the dominant role of panic disorder, anxiety, and sleep deprivation suggests that mental health support and sleep health interventions should be central—not secondary—components of occupational health programmes. Third, the distinct associations of tobacco, ENDS, and dual use with chest pain subtypes indicate that all nicotine products should be targeted in cessation counselling, rather than focusing solely on conventional tobacco. Finally, the associations with seniority and country income point to the need for institution-level and policy-level strategies, particularly for senior clinicians and those working in lower-resource health systems.

Beyond immediate practice, these findings also identify clear priorities for future research. Prospective studies incorporating objective cardiac endpoints — including electrocardiography, biomarkers, and imaging — are needed to determine whether the symptom burden observed here translates into measurable cardiovascular risk over time. Intervention studies are also required to establish whether targeted improvements in sleep and psychological health reduce chest pain burden in this workforce, and whether such interventions are feasible within the constraints of clinical training and practice. Finally, the observed differences by country income level highlight the need for health-system-level research examining how staffing, workload, and access to occupational health services shape symptom burden across diverse healthcare settings

### Strengths and limitations

This study has several strengths. It represents one of the largest multinational investigations of chest pain among physicians, including 6,224 physicians from 12 countries in the MENA region, providing substantial statistical power and enhancing the robustness of the findings. The use of standardized instruments to assess chest pain, anxiety, and panic disorders, along with the application of multivariable regression models, strengthens the internal validity of the results.

However, several limitations should be acknowledged. The cross-sectional design precludes conclusions about temporality or causality. Chest pain classification was based on the self-reported RAQ rather than clinical evaluation, electrocardiography, or biomarkers, and therefore cannot definitively distinguish ischemic from non-ischemic causes. This limitation is particularly salient given the high prevalence of panic disorder in our cohort: panic attacks can be precipitated by exertion and subside with rest, mimicking the exact RAQ criteria for definitive anginal pain. This overlap may partly explain the stronger association of panic disorder with definitive than possible anginal pain and suggests RAQ specificity is attenuated in populations with high panic disorder prevalence. The very high prevalence of possible anginal pain likely includes a heterogeneous mix of gastrointestinal, musculoskeletal, respiratory, and psychogenic etiologies. All exposures were self-reported, introducing the possibility of recall bias and social desirability bias, particularly for psychological disorders and substance use. The screen-positive status of anxiety disorder and panic disorder may over- or under-estimate clinically diagnosed prevalence; no gold-standard diagnostic measure exists. Although the sample was large and multinational, online recruitment may have introduced selection bias toward more engaged or digitally connected physicians. As Egypt, Iraq, Yemen, and Jordan contributed 67.3% of the sample, Egypt alone 29.0%, the country-income findings largely reflect these four settings rather than the MENA region as a whole, and country-level analysis was precluded for underrepresented countries. Moreover, the study was restricted to physicians aged 24–60 years; therefore, the results may not generalise beyond this range. Finally, residual confounding from unmeasured variables—such as family history, dietary patterns, or specific occupational exposures—cannot be excluded.

### Conclusion

Chest pain was highly prevalent among physicians in this large MENA-region cohort, with the pattern of determinants suggesting that psychological, occupational, and behavioral factors — particularly panic disorder, anxiety, sleep deprivation, obesity, and nicotine product use — were strongly associated with chest pain alongside traditional cardiovascular risk factors. While the symptom profiles and risk factor patterns are consistent with a substantial non-cardiac contribution in this relatively young professional population, the prevalence of definitive anginal pain and the associations with tobacco use and obesity indicate that true cardiovascular pathology cannot be discounted. These findings call for an integrated occupational health response that addresses both psychological and cardiovascular risk, incorporating mental health support, sleep health interventions, and nicotine cessation alongside cardiac surveillance. Longitudinal studies with objective clinical endpoints are needed to better characterize the long-term cardiovascular consequences of this symptom burden.

## Supporting information

S1 Table Sample distribution. (PDF)

S2 Table STROBE Statement—Checklist of items that should be included in reports of cross-sectional studies. (PDF)

S3 Table Univariate binomial regression analysis for individual variables with chest pain (combined possible and definite anginal chest pain) as the dependent variable, and never experienced chest pain as the reference. (PDF)

S4 Table Univariate multinomial regression analysis for individual variables with chest pain (possible vs definite anginal chest pain) as the dependent variable, and never experienced chest pain as the reference. (PDF)

S1 File Author-developed questionnaire items. (PDF)

S2 File Fully de-identified minimal individual-level dataset underlying the study analyses. (XLSX)

S3 File PLOS’ questionnaire on inclusivity in global research. (PDF)
