# Supplementary material for: Prevalence, characteristics, and determinants of chest pain among physicians in the Middle East and North Africa: A multinational cross-sectional study
Source: PLoS One. 2026 Aug 31;21(8):e0355946. doi: 10.1371/journal.pone.0355946 (PMC13529011; doi:10.1371/journal.pone.0355946)
Supplement: Supplementary file 1 [file pone.0355946.s001.pdf]

Supplementary Table 1

Supplementary Table 1. Sample distribution

Country of residence	Experienced definitive anginal pain (N=795)	Experienced possible anginal pain (N=2836)	Never experienced chest pain (N=2593)	Total (N=6224)
Egypt	268.0 (33.7%)	880.0 (31.0%)	658.0 (25.4%)	1806.0 (29.0%)
Iraq	114.0 (14.3%)	472.0 (16.6%)	317.0 (12.2%)	903.0 (14.5%)
Jordan	80.0 (10.1%)	323.0 (11.4%)	300.0 (11.6%)	703.0 (11.3%)
Libya	20.0 (2.5%)	132.0 (4.7%)	132.0 (5.1%)	284.0 (4.6%)
Morocco	12.0 (1.5%)	47.0 (1.7%)	30.0 (1.2%)	89.0 (1.4%)
Palestine (West Bank and Gaza)	38.0 (4.8%)	103.0 (3.6%)	94.0 (3.6%)	235.0 (3.8%)
Qatar	29.0 (3.6%)	74.0 (2.6%)	130.0 (5.0%)	233.0 (3.7%)
Saudi Arabia	38.0 (4.8%)	111.0 (3.9%)	147.0 (5.7%)	296.0 (4.8%)
Sudan	56.0 (7.0%)	146.0 (5.1%)	207.0 (8.0%)	409.0 (6.6%)
Syria	44.0 (5.5%)	180.0 (6.3%)	192.0 (7.4%)	416.0 (6.7%)
United Arab Emirates	10.0 (1.3%)	26.0 (0.9%)	34.0 (1.3%)	70.0 (1.1%)
Yemen	86.0 (10.8%)	342.0 (12.1%)	352.0 (13.6%)	780.0 (12.5%)
