# Supplementary material for: Prevalence, characteristics, and determinants of chest pain among physicians in the Middle East and North Africa: A multinational cross-sectional study
Source: PLoS One. 2026 Aug 31;21(8):e0355946. doi: 10.1371/journal.pone.0355946 (PMC13529011; doi:10.1371/journal.pone.0355946)
Supplement: Supplementary file 3 [file pone.0355946.s003.pdf]

**Supplementary Table 3**

**Supplementary Table 3. Univariate binomial regression analysis for individual variables with chest pain (combined possible and definite anginal chest pain) as the dependent variable, and never experienced chest pain as the reference**

| Variable | OR (95% CI) | p-value |
|---|---|---|
| Age (years) | 1.00 (0.99–1.01) | 0.71 |
| Sex (reference: male) | 1.41 (1.27–1.56) | <0.001 |
| Marital status (reference: single) | | |
| Married | 1.08 (0.97–1.20) | 0.156 |
| Separated | 2.21 (1.04–4.72) | 0.04 |
| Divorced | 1.44 (0.84–2.48) | 0.188 |
| Widowed | 1.03 (0.33–3.26) | 0.955 |
| Body mass index (reference: healthy weight) | | |
| Underweight | 1.24 (0.93–1.64) | 0.144 |
| Overweight | 1.08 (0.96–1.20) | 0.197 |
| Obese | 1.39 (1.20–1.62) | <0.001 |
| Exercise frequency (reference: non-exercising) | | |
| 1–2 times/week | 1.08 (0.95–1.22) | 0.246 |
| 3–5 times/week | 0.88 (0.77–1.00) | 0.057 |
| >5 times/week | 1.02 (0.75–1.38) | 0.921 |
| Physical comorbidities (reference: no) | 2.10 (1.77–2.49) | <0.001 |
| Psychiatric comorbidities (reference: no) | 2.33 (1.74–3.12) | <0.001 |
| Sleep deprivation (reference: no) | 2.12 (1.91–2.36) | <0.001 |
| Anxiety disorder (reference: no) | 2.18 (1.96–2.43) | <0.001 |
| Panic disorder (reference: no) | 3.29 (2.93–3.69) | <0.001 |
| Nicotine product use (reference: non-users) | | |
| Exclusive ENDS users | 1.29 (1.03–1.61) | 0.025 |
| Exclusive tobacco users | 1.29 (1.08–1.55) | 0.006 |
| Dual users | 1.65 (1.19–2.29) | 0.002 |
| Caffeinated drinks consumption (reference: no) | 1.03 (0.87–1.22) | 0.74 |
| Alcohol consumption (reference: no) | 1.35 (1.01–1.81) | 0.042 |
| Recreational drug use (reference: no) | 1.27 (0.97–1.67) | 0.078 |
| Academic/professional degree (reference: Bachelor’s) | | |
| Diploma | 1.02 (0.74–1.40) | 0.929 |
| Fellowship/board | 1.20 (0.98–1.49) | 0.085 |
| Master’s | 1.00 (0.83–1.20) | 0.996 |
| Doctoral | 1.24 (0.93–1.64) | 0.145 |
| Seniority (reference: house officer) | | |
| General practitioner | 1.18 (1.03–1.35) | 0.018 |
| Resident | 1.26 (1.10–1.44) | <0.001 |

| | | |
|---|---|---|
| Attending | 1.24 (1.05–1.48) | 0.012 |
| Years of experience | 1.01 (1.00–1.02) | 0.023 |
| Working hours/week (reference: <40 hours/week) | | |
| 40–80 hours/week | 1.04 (0.94–1.15) | 0.47 |
| >80 hours/week | 1.29 (1.04–1.59) | 0.019 |
| Country income level (reference: high) | | |
| Low | 1.23 (1.02–1.48) | 0.032 |
| Middle | 1.76 (1.48–2.09) | <0.001 |

Abbreviations: OR, odds ratio; 95% CI, 95% confidence interval; ENDS, electronic nicotine delivery system.
