# Supplementary material for: Prevalence, characteristics, and determinants of chest pain among physicians in the Middle East and North Africa: A multinational cross-sectional study
Source: PLoS One. 2026 Aug 31;21(8):e0355946. doi: 10.1371/journal.pone.0355946 (PMC13529011; doi:10.1371/journal.pone.0355946)
Supplement: Supplementary file 4 [file pone.0355946.s004.pdf]

Supplementary Table 4

Supplementary Table 4. Univariate multinomial regression analysis for individual variables with chest pain (possible vs definite anginal chest pain) as the dependent variable, and never experienced chest pain as the reference

Variable	Definitive anginal pain		Possible anginal pain	
	OR (95% CI)	p-value	OR (95% CI)	p-value
Age (years)	1.01 (0.99–1.02)	0.501	1.00 (0.99–1.01)	0.862
Sex (reference: male)	1.91 (1.63–2.25)	<0.001	1.29 (1.16–1.44)	<0.001
Marital status (reference: single)				
Married	1.21 (1.02–1.43)	0.026	1.05 (0.93–1.17)	0.434
Separated	1.56 (0.48–5.10)	0.457	2.40 (1.11–5.20)	0.027
Divorced	1.75 (0.82–3.77)	0.150	1.35 (0.76–2.40)	0.300
Widowed	-	-	1.31 (0.42–4.15)	0.642
Body mass index (reference: healthy weight)				
Underweight	1.42 (0.93–2.18)	0.105	1.19 (0.88–1.60)	0.267
Overweight	1.20 (1.00–1.43)	0.048	1.05 (0.93–1.18)	0.469
Obese	1.34 (1.06–1.70)	0.015	1.41 (1.20–1.65)	<0.001
Exercise frequency (reference: non-exercising)				
1–2 times/week	0.98 (0.81–1.19)	0.862	1.11 (0.97–1.26)	0.136
3–5 times/week	0.67 (0.54–0.84)	<0.001	0.94 (0.82–1.08)	0.397
>5 times/week	1.15 (0.74–1.80)	0.538	0.97 (0.70–1.35)	0.876
Physical comorbidities (reference: no)	1.80 (1.40–2.32)	<0.001	2.19 (1.83–2.61)	<0.001
Psychiatric comorbidities (reference: no)	1.91 (1.25–2.92)	0.003	2.45 (1.81–3.31)	<0.001
Sleep deprivation (reference: no)	2.52 (2.14–2.96)	<0.001	2.03 (1.82–2.26)	<0.001
Anxiety disorder (reference: no)	2.46 (2.09–2.90)	<0.001	2.11 (1.88–2.37)	<0.001
Panic disorder (reference: no)	4.38 (3.70–5.19)	<0.001	3.03 (2.69–3.42)	<0.001
Nicotine product use (reference: non-users)				
Exclusive ENDS users	1.12 (0.79–1.59)	0.529	1.34 (1.06–1.68)	0.014
Exclusive tobacco users	1.36 (1.04–1.79)	0.024	1.27 (1.05–1.54)	0.014

Dual users	1.13 (0.66–1.94)	0.661	1.81 (1.29–2.52)	<0.001
Caffeinated drinks consumption (reference: no)	1.15 (0.87–1.52)	0.316	1.00 (0.84–1.19)	0.988
Alcohol consumption (reference: no)	1.19 (0.75–1.87)	0.465	1.40 (1.03–1.89)	0.030
Recreational drug use (reference: no)	1.34 (0.90–2.00)	0.151	1.26 (0.95–1.67)	0.117
Academic/professional degree (reference: Bachelor’s)				
Diploma	1.18 (0.73–1.92)	0.495	0.97 (0.69–1.37)	0.855
Fellowship/board	1.06 (0.76–1.49)	0.722	1.24 (1.00–1.55)	0.054
Master’s	1.24 (0.94–1.62)	0.128	0.94 (0.77–1.14)	0.504
Doctoral	1.42 (0.94–2.15)	0.1	1.19 (0.88–1.60)	0.269
Seniority (reference: house officer)				
General practitioner	1.05 (0.85–1.30)	0.637	1.22 (1.05–1.40)	0.008
Resident	1.10 (0.89–1.36)	0.394	1.30 (1.13–1.50)	<0.001
Attending	1.29 (1.00–1.67)	0.055	1.23 (1.03–1.48)	0.025
Years of experience	1.02 (1.00–1.03)	0.072	1.01 (1.00–1.02)	0.042
Working hours/week (reference: <40 hours/week)				
40–80 hours/week	1.00 (0.85–1.18)	0.968	1.05 (0.94–1.17)	0.39
>80 hours/week	1.16 (0.83–1.61)	0.392	1.33 (1.06–1.66)	0.012
Country income level (reference: high)				
Low	1.00 (0.74–1.35)	0.998	1.31 (1.07–1.61)	0.009
Middle	1.40 (1.07–1.84)	0.013	1.88 (1.56–2.27)	<0.001

Abbreviations: OR, odds ratio; 95% CI, 95% confidence interval; ENDS, electronic nicotine delivery system
