# Supplementary material for: Prevalence, characteristics, and determinants of chest pain among physicians in the Middle East and North Africa: A multinational cross-sectional study
Source: PLoS One. 2026 Aug 31;21(8):e0355946. doi: 10.1371/journal.pone.0355946 (PMC13529011; doi:10.1371/journal.pone.0355946)
Supplement: Supplementary file 5 [file pone.0355946.s005.pdf]

Author-developed questionnaire items

This file reproduces the author-developed items of the study survey. The standardized, validated instruments embedded in the survey — the Rose Angina Questionnaire (RAQ), the Generalized Anxiety Disorder-7 (GAD-7) scale, and the Panic Disorder Screener (PADIS) — are described and cited in the main text and are not reproduced here. The questionnaire was administered in English via an online form.

Personal data

1. Age
2. Sex
3. Marital status
4. Do you have children?
5. Weight
6. Height
7. Do you suffer from sleep deprivation most of your days?
8. Do you have any physical illness? (If yes, please specify.)
9. Do you have any mental illness? (If yes, please specify.)
10. Do you smoke tobacco or use e-cigarettes (vape)? *
11. For how many years have you smoked tobacco or used e-cigarettes (vape)?
12. If you smoke cigarettes, how many cigarettes do you smoke per day on average? *
13. Do you use recreational drugs?
14. Do you drink coffee or tea?
15. Do you drink alcohol?
16. How many times do you exercise per week?
17. Highest educational degree
18. Seniority rank
19. Specialty
20. Place of work
21. Number of years employed in the role (years of experience)
22. Average working hours per week
23. Nationality
24. Country of residence
