# Supplementary material for: Prevalence, characteristics, and determinants of chest pain among physicians in the Middle East and North Africa: A multinational cross-sectional study
Source: PLoS One. 2026 Aug 31;21(8):e0355946. doi: 10.1371/journal.pone.0355946 (PMC13529011; doi:10.1371/journal.pone.0355946)
Supplement: Supplementary file 7 [file pone.0355946.s008.pdf]

PLOS One

Prevalence, characteristics, and determinants of chest pain among physicians in the Middle East and North Africa: A multinational cross-sectional study

--Manuscript Draft--

Manuscript Number:	PONE-D-26-19587
Article Type:	Research Article
Full Title:	Prevalence, characteristics, and determinants of chest pain among physicians in the Middle East and North Africa: A multinational cross-sectional study
Short Title:	Chest pain among physicians in the MENA region
Corresponding Author:	Weam Aldiban, Ph.D<br>International University for Science and Technology College of Pharmacy<br>Damascus, SYRIAN ARAB REPUBLIC
Keywords:	chest pain; Angina; Physicians; Middle East and North Africa; Cross-sectional study; Rose Angina Questionnaire
Abstract:	Background: Physicians face substantial occupational, psychological, and cardiovascular risk factors predisposing them to chest pain, yet no large-scale study has examined this in the Middle East and North Africa (MENA). This study determined the prevalence, characteristics, and determinants of chest pain among MENA physicians.<br>Methods: This multinational cross-sectional study enrolled 6,224 physicians from 12 MENA countries (August–December 2023). Chest pain was classified using the Rose Angina Questionnaire. Anxiety and panic disorder were assessed using the Generalized Anxiety Disorder-7 (GAD-7) and Panic Disorder Screener (PADIS). Binary and multinomial logistic regression identified independent determinants.<br>Results: Overall, 795 (12.8%) met criteria for definitive anginal pain and 2,836 (45.6%) for possible anginal pain. In the adjusted binary model, panic disorder was the strongest predictor (AOR = 2.76, 95% CI: 2.44–3.13), followed by physical comorbidities (AOR = 1.91), sleep deprivation (AOR = 1.61), female sex (AOR = 1.49), anxiety disorder (AOR = 1.38), and obesity (AOR = 1.30). All nicotine product categories were independently associated with chest pain. Multinomial analysis revealed stronger associations of panic disorder (AOR = 3.52) and tobacco use with definitive anginal pain, while ENDS use was significant only for possible anginal pain. Middle-income country physicians had higher odds than those from high-income countries (AOR = 1.71).<br>Conclusion: Chest pain is highly prevalent among MENA physicians, driven primarily by panic disorder, anxiety, sleep deprivation, and nicotine product use. Integrated occupational health programmes addressing both psychological and cardiovascular risk are urgently needed.
Order of Authors:	Saadi M. Saleh
	Weam Aldiban, Ph.D
	Alaa Aljamala
	Khaled Gadallah
	Omar Habib
	Mansour Algazar
	Omar Abdalazeez
	Safia Abdi
	Noorulhuda Oudah
	Hamza Alhamdan
	Bushra M. Rageh
	Rasha Shaker Eldesouky

Powered by Editorial Manager® and ProduXion Manager® from Aries Systems Corporation

Additional Information:	
**Question**	**Response**
**Financial Disclosure**<br><br>Enter a financial disclosure statement that describes the sources of funding for the work included in this submission. Review the submission guidelines for detailed requirements. View published research articles from *PLOS ONE* for specific examples.<br><br>This statement is required for submission and **will appear in the published article** if the submission is accepted. Please make sure it is accurate.<br><br>**Funded studies**<br>Enter a statement with the following details:<br>• Initials of the authors who received each award<br>• Grant numbers awarded to each author<br>• The full name of each funder<br>• URL of each funder website<br>• Did the sponsors or funders play any role in the study design, data collection and analysis, decision to publish, or preparation of the manuscript?<br><br>Did you receive funding for this work?	The author(s) received no specific funding for this work.
**Competing Interests**<br><br>Use the instructions below to enter a competing interest statement for this submission. On behalf of all authors, disclose any competing interests that could be perceived to bias this work—acknowledging all financial support and any other relevant financial or non-financial competing interests.<br><br>This statement is required for submission and **will appear in the published article** if the submission is accepted. Please make sure it is accurate and that any funding sources listed in your Funding Information	The authors have declared that no competing interests exist.

later in the submission form are also declared in your Financial Disclosure statement.<br><br>View published research articles from *PLOS ONE* for specific examples.<br><br>**NO authors have competing interests**<br><br>Enter: *The authors have declared that no competing interests exist.*<br><br>**Authors with competing interests**<br><br>Enter competing interest details beginning with this statement:<br><br>*I have read the journal's policy and the authors of this manuscript have the following competing interests: [insert competing interests here]*<br><br>* typeset	
**Ethics Statement**<br><br>Enter an ethics statement for this submission. This statement is required if the study involved:<br><br>• Human participants<br>• Human specimens or tissue<br>• Vertebrate animals or cephalopods<br>• Vertebrate embryos or tissues<br>• Field research<br><br>Write "N/A" if the submission does not require an ethics statement.<br><br>General guidance is provided below. Consult the submission guidelines for detailed instructions. **Make sure that all information entered here is included in the Methods section of the manuscript.**	The study received ethical approval from the Research Ethics Committee at the Faculty of Medicine, Benha University (REC-FOMBU), Egypt (registration number: RC.12.8.2023), and from Alrazi University, Sana'a City, Yemen (reference number: 016/FMHS/2023)

**Format for specific study types**<br><br>**Human Subject Research (involving human participants and/or tissue)**<br>• Give the name of the institutional review board or ethics committee that approved the study<br>• Include the approval number and/or a statement indicating approval of this research<br>• Indicate the form of consent obtained (written/oral) or the reason that consent was not obtained (e.g. the data were analyzed anonymously)<br><br>**Animal Research (involving vertebrate animals, embryos or tissues)**<br>• Provide the name of the Institutional Animal Care and Use Committee (IACUC) or other relevant ethics board that reviewed the study protocol, and indicate whether they approved this research or granted a formal waiver of ethical approval<br>• Include an approval number if one was obtained<br>• If the study involved *non-human primates*, add *additional details* about animal welfare and steps taken to ameliorate suffering<br>• If anesthesia, euthanasia, or any kind of animal sacrifice is part of the study, include briefly which substances and/or methods were applied<br><br>**Field Research**<br><br>Include the following details if this study involves the collection of plant, animal, or other materials from a natural setting:<br>• Field permit number<br>• Name of the institution or relevant body that granted permission	
Data Availability<br><br>Before publication, Authors are required to make fully available and without restriction all data underlying their findings. Sharing the data underlying your findings increases the visibility, credibility, and impact of your work, while supporting transparency and future scientific discovery. Please see our PLOS Data Policy page for detailed information on this policy.</p><br>A Data Availability	All data available as request from corresponding author

Statement, detailing where the data can be accessed, is required at first submission. Insert your Data Availability Statement in the box below.</p><br><br>Please see the data reporting of our submission guidelines for instructions on what you need to include in your Data Availability Statement. Examples of acceptable Data Availability Statements are included here for reference:</p><br> <br> - The minimal data set [and accompanying code, where generated] is available at [repository name] via [DOI hyperlink] <br> - No data was generated by this study. The following existing data sources were used: [dataset name] from [dataset location] available via [web address or DOI hyperlink]. <br> - For Study Protocols: No datasets were generated or analysed during the current study. All relevant data from this study will be made available upon study completion. <br> <br><br><i>PLOS allows rare exemptions to address legal and ethical concerns. If you have legal or ethical restrictions, please use the box below to detail these in full sentences for the Journal team to consider.</i></p><br><br>This statement will be typeset if the manuscript is accepted for publication.</p>	

Powered by Editorial Manager® and ProduXion Manager® from Aries Systems Corporation

Manuscript

Click here to access/download;Manuscript;Manuscript Plos One (1).docx

Prevalence, characteristics, and determinants of chest pain among physicians in the Middle East and North Africa: A multinational cross-sectional study

Short Title: Chest pain among physicians in the MENA region

Saadi M. Saleh[1, 2]; Weam Aldiban[3 †, *]; Alaa Aljamala[2, †]; Khaled Gadallah[4]; Omar Habib[5]; Mansour Algazar[6]; Omar Abdalazeez[7]; Safia Abdi[8]; Noorulhuda Oudah[9] ; Hamza Alhamdan[10]; Bushra M. Rageh[11]; DARS Consortium[12]; Rasha Shaker Eldesouky[13]

1. Department of Internal Medicine, Hamad Medical Corporation, Doha, Qatar

2. Faculty of Medicine, Benha University, Benha, Egypt

3. Faculty of Pharmacy, International University for Science and Technology (IUST), Daraa, Syrian Arab Republic

4. Faculty of Medicine, Menoufia University, Menoufia, Egypt

5. Faculty of Medicine, Portsaid University, Portsaid, Egypt

6. Faculty of Medicine, Alazhar University, Asyut, Egypt

7. Faculty of Medicine, Alazhar University, Cairo, Egypt

8. Omdurman Islamic University Faculty of Medicine, Khartoum, Sudan

9. Alkindy College of Medicine, Baghdad University, Baghdad, Iraq

10. Hashemite University Faculty of Medicine, Zarqa, Jordan

11. Department of Physiology, Faculty of Medicine, Sana'a University, Sana'a, Yemen

12. Dijla Academic Research Students (DARS) Consortium

13. Vice Dean of Education and Student Affairs, Faculty of Medicine, Benha University, Banha, Egypt.

† These authors contributed equally to this work

***Corresponding Author:** Weam Aldiban; Faculty of Pharmacy, International University for Science and Technology (IUST), Daraa, Syrian Arab Republic; Email: weam.aldiban@iust.edu.sy; Phone Number: +963-966231723

Abstract

Background: Physicians face substantial occupational, psychological, and cardiovascular risk factors predisposing them to chest pain, yet no large-scale study has examined this in the Middle East and North Africa (MENA). This study determined the prevalence, characteristics, and determinants of chest pain among MENA physicians.

Methods: This multinational cross-sectional study enrolled 6,224 physicians from 12 MENA countries (August–December 2023). Chest pain was classified using the Rose Angina Questionnaire. Anxiety and panic disorder were assessed using the Generalized Anxiety Disorder-7 (GAD-7) and Panic Disorder Screener (PADIS). Binary and multinomial logistic regression identified independent determinants.

Results: Overall, 795 (12.8%) met criteria for definitive anginal pain and 2,836 (45.6%) for possible anginal pain. In the adjusted binary model, panic disorder was the strongest predictor (AOR = 2.76, 95% CI: 2.44–3.13), followed by physical comorbidities (AOR = 1.91), sleep deprivation (AOR = 1.61), female sex (AOR = 1.49), anxiety disorder (AOR = 1.38), and obesity (AOR = 1.30). All nicotine product categories were independently associated with chest pain. Multinomial analysis revealed stronger associations of panic disorder (AOR = 3.52) and tobacco use with definitive anginal pain, while ENDS use was significant only for possible anginal pain. Middle-income country physicians had higher odds than those from high-income countries (AOR = 1.71).

Conclusion: Chest pain is highly prevalent among MENA physicians, driven primarily by panic disorder, anxiety, sleep deprivation, and nicotine product use. Integrated occupational health programmes addressing both psychological and cardiovascular risk are urgently needed.

Keywords

Chest pain; Angina; Physicians; Middle East and North Africa; Cross-sectional study; Rose Angina Questionnaire

Introduction

Chest pain is among the most clinically significant and diagnostically complex symptoms encountered in medical practice. It accounts for approximately 6–8% of all emergency department visits globally and represents a leading cause of unscheduled healthcare utilization (1,2). While chest pain carries an important association with serious cardiovascular conditions — including acute coronary syndromes, stable angina pectoris, and aortic disease — the majority of chest pain presentations in community and outpatient settings are non-cardiac in origin, arising from gastrointestinal, musculoskeletal, pulmonary, or psychological causes (3–5). This diagnostic complexity is compounded by the substantial overlap between cardiac and non-cardiac presentations, particularly in the context of anxiety disorders and panic disorder, which can produce chest pain that closely mimics classical angina both clinically and experientially (6,7).

The Middle East and North Africa (MENA) regions present a distinctive epidemiological context for cardiovascular disease. Cardiovascular events in this region occur at younger ages than in Western counterparts, driven by a rapidly increasing prevalence of cardiovascular risk factors including obesity, type 2 diabetes, dyslipidaemia, and tobacco use (8,9). Despite this growing burden, population-level data on chest pain symptoms — as distinct from established cardiovascular disease endpoints — remain sparse, and the determinants of chest pain in this region are poorly characterised.

Healthcare workers constitute one of the most occupationally stressed professional groups globally. The demands of clinical practice — encompassing prolonged working hours, shift

work, night duties, emotional labour, exposure to patient suffering and death, and the perpetual pressure of clinical decision-making — impose a substantial and sustained physiological burden on those who deliver care, one that is increasingly reflected in elevated rates of cardiovascular symptoms including chest pain (10,11). Growing attention has also been directed toward broader downstream physical health consequences of occupational stress in this group, including musculoskeletal disorders, metabolic dysfunction, and cardiovascular disease (10,12).

Healthcare workers in the MENA region face a dual vulnerability: they are exposed to the same population-level cardiovascular risk factors as the general public, while simultaneously bearing the disproportionate physiological consequences of demanding occupational environments. Furthermore, psychological comorbidities — including anxiety disorders, panic disorder, and depression — are prevalent in this workforce and are increasingly recognised as independent contributors to both cardiac and non-cardiac chest pain through mechanisms including chronic autonomic dysregulation, systemic inflammation, and heightened somatic symptom perception (7,13,14). Moreover, the region's healthcare systems span a wide range of socioeconomic development levels, from high-income Gulf states to lower-middle-income countries, creating substantial heterogeneity in working conditions, staffing ratios, and access to occupational health support — all of which may meaningfully influence chest pain risk (8,15).

Despite this convergence of occupational, psychological, and cardiovascular risk factors, no large-scale study has examined the prevalence and determinants of chest pain specifically among healthcare workers in the MENA region. Existing studies from the region have focused predominantly on cardiovascular disease endpoints in the general population (8,9), while the occupational health literature on MENA healthcare workers has centred on burnout, mental

health, and infection-related exposures (13,16,17). Therefore, this study aimed to determine the prevalence, clinical characteristics, and determinants of chest pain among physicians in the MENA region.

Methods

Study design, setting, and participants

This is a multinational cross-sectional study conducted among physicians from 12 MENA countries between August 25 and December 25, 2023 (Supplementary Table 1). Participants were eligible if they were physicians actively practising medicine within the MENA region and aged between 24 and 60 years. Only respondents who met both eligibility criteria were included in the analysis. The study was reported in accordance with the Strengthening the Reporting of Observational Studies in Epidemiology (STROBE) checklist (Supplementary Table 2) (18).

Ethical considerations and approval

The study was conducted in accordance with the principles of the Declaration of Helsinki (19), and received ethical approval from the Research Ethics Committee at the Faculty of Medicine, Benha University (REC-FOMBU), Egypt (registration number: RC.12.8.2023), and from Alrazi University, Sana'a City, Yemen (reference number: 016/FMHS/2023). Each participant was provided with a cover page detailing the study's purpose and containing statements regarding confidentiality, anonymity, voluntary participation, and data privacy. Completion of the questionnaire was contingent on providing electronic informed consent, and participants retained the right to withdraw at any stage without consequence.

Data collection tools and procedures

Data were collected using an English-language self-administered online questionnaire developed via Google Forms (Google LLC). The survey was disseminated through a network of

collaborators across participating countries via social media platforms, including Facebook, Telegram, WhatsApp, Twitter, and Snapchat. Collaborators facilitated distribution within relevant academic and professional communities, and participants were screened for eligibility prior to receiving access to the survey link. To ensure data integrity, the survey was configured to permit only one response per participant. Responses were additionally reviewed manually, and those deemed invalid — including entries with repetitive or identical answer patterns — were excluded from the final analysis.

The survey comprised four sections. The first section collected sociodemographic information, including age, sex, marital status, country of residence, and nationality, alongside social history (nicotine product use, alcohol consumption, recreational drug use, and caffeinated drinks consumption). Occupational variables included seniority rank, medical specialty, place of work, years of experience, and working hours per week. Health-related variables included self-reported height and weight for body mass index (BMI) calculation, subjective sleep deprivation, physical comorbidities, and psychiatric comorbidities. The country income level variable was created by grouping the participating countries into high, middle, and low income according to the World Bank 2023 income classification (20,21).

The second section contained the Rose Angina Questionnaire (RAQ), a validated epidemiological instrument for classifying chest pain based on standardised symptom criteria (22). First developed in 1962, the RAQ has been used extensively in multinational epidemiological studies and translated into nine languages (23). It demonstrates high specificity (80–95%) with variable sensitivity (19–83%) depending on the reference standard applied (23,24). The RAQ includes structured questions assessing the presence, location, character, and

triggers of chest pain, and its relationship to physical exertion and rest. Chest pain was classified into three categories: no chest pain, possible anginal pain, and definitive anginal pain. Participants were classified as having no chest pain if they reported never experiencing chest pain or discomfort. Definitive anginal pain was defined as chest pain precipitated by exertion (walking uphill, hurrying, or walking at an ordinary pace), causing the participant to stop or slow down, relieved by rest within 10 minutes, and localised either to the sternum (any level) or the left arm and left anterior chest. Participants reporting chest pain that did not fulfil all these criteria were classified as having possible anginal pain.

The third section assessed anxiety symptoms using the Generalized Anxiety Disorder-7 (GAD-7) questionnaire, a validated seven-item self-administered screening tool. Each item is rated on a four-point Likert scale from 0 (“not at all”) to 3 (“nearly every day”), yielding a total score of 0 to 21. A cut-off score of ≥10 was used to define clinically significant anxiety, consistent with validation studies reporting sensitivity of 89% and specificity of 82% at this threshold (25).

The fourth section evaluated panic disorder using the Panic Disorder Screener (PADIS), a brief validated instrument designed for community-based screening. The PADIS comprises four items: an initial screening question assessing the frequency of panic attacks in the past month (scored 0–4 on a five-point Likert scale), followed by three items evaluating associated symptoms and functional impact (each scored 0–3), yielding a total score of 0 to 13. Participants who reported no panic attacks on the initial item were not required to complete the remaining items, and their scores were recorded as zero. A cut-off score of ≥4 was used to indicate the presence of panic disorder, corresponding to a sensitivity of 77% and specificity of 84% (26).

Sample size and sampling

The minimum required sample size was calculated a priori using the Scalex SP calculator (27), based on an expected prevalence of chest pain of 3.5%, as reported by Wang et al. (28). A minimum of 424 participants was required to estimate this prevalence with an absolute precision of ±1.75% and 95% confidence, yielding an anticipated confidence interval of 1.75% to 5.25%. Participants were recruited using convenience sampling. The final analytic sample of 6,224 participants substantially exceeded the minimum required size, conferring greater statistical precision and power than originally planned, and providing sufficient power for the multivariable binary and multinomial logistic regression analyses across 12 countries and multiple covariates (29).

Statistical analysis

All statistical analyses were performed using jamovi (version 2.3; The jamovi Project, Sydney, Australia). The survey was configured with mandatory fields, so no missing data occurred. Continuous variables were summarised as means with standard deviations (SD), and categorical variables as frequencies and percentages. Between-group differences across the three chest pain categories were examined using one-way analysis of variance (ANOVA) for continuous variables and Pearson's chi-squared test for categorical variables.

To identify factors independently associated with chest pain, a binary logistic regression analysis was conducted with chest pain (combining definitive and possible anginal pain) as the dependent variable, and no chest pain as the reference category. Crude and adjusted odds ratios (OR and AOR) with corresponding 95% confidence intervals (CI) and *p*-values were reported. Covariates were selected for inclusion in the initial adjusted model based on a combination of clinical relevance and statistical significance in univariate analysis ($p < 0.20$); those that were non-

significant and did not improve model fit were removed from the final model. The adjusted binary model included the following variables: age, sex, body mass index category, physical comorbidities, subjective sleep deprivation, anxiety disorder, panic disorder, nicotine product use, seniority rank, and country income level. Multicollinearity among independent variables was assessed using variance inflation factors (VIF), with all values indicating no significant collinearity ($VIF < 5$). Model performance for the binary logistic regression model was evaluated using Akaike Information Criterion (AIC), McFadden's pseudo-R^2, and area under the receiver operating characteristic curve (AUC), along with sensitivity, specificity, and overall classification accuracy.

A sensitivity analysis was conducted by additionally including psychiatric comorbidities in the adjusted binary model to assess the robustness of the primary estimates against residual confounding from overlapping psychological variables.

To examine whether the determinants of chest pain differed between subtypes, multinomial logistic regression analysis was performed with chest pain as a three-level outcome — definitive anginal pain, possible anginal pain, and no chest pain — using the no-chest-pain group as the reference category. This approach allowed simultaneous estimation of subtype-specific associations, providing greater epidemiological granularity than binary modelling alone. Adjusted odds ratios with 95% CIs and *p*-values were reported for each outcome category. Variable selection followed the same criteria as the binary model, except that seniority rank was not retained in the adjusted multinomial model as it did not reach statistical significance for either chest pain outcome category. Model fit was assessed using AIC and McFadden's pseudo-R^2.

Medical specialty was evaluated as a candidate covariate but excluded from the final models because of instability caused by sparse cells across 23 specialty categories.

To enhance transparency and reproducibility, complete univariate results for all candidate variables — including those not retained in the adjusted models — are presented in Supplementary Tables 3 and 4 for the binary and multinomial analyses, respectively. All reported *p*-values are two-tailed. Odds ratios and confidence interval bounds are presented rounded to two decimal places, consistent with standard epidemiological reporting practice. No adjustments were made for multiple comparisons, as the analyses were hypothesis-driven and based on pre-specified clinically relevant variables rather than exploratory data mining.

Results

Sample characteristics

A total of 6,224 physicians were included in the analysis. Of these, 795 (12.8%) reported experiencing definitive anginal pain, 2,836 (45.6%) reported possible anginal pain, and 2,593 (41.7%) had never experienced chest pain. The characteristics of the study participants are presented in Table 1.

The mean age of participants was 29.1 years (SD = 5.9), with no statistically significant difference across the three groups (p = 0.797). The majority were males (55.4%), singles (65.3%), and held a bachelor's degree (79.4%). The mean BMI was 25.7 kg/m^2 (SD = 5.0), with a statistically significant difference across groups (p = 0.016). Obesity was more prevalent among those with possible anginal pain (17.6%) compared to those who never experienced chest pain (13.5%) (p < 0.001).

Regarding mental health and sleep, subjective sleep deprivation was reported by 55.3% of participants with definitive anginal pain, 50.0% with possible anginal pain, and 33.0% of those who never experienced chest pain (p < 0.001). Anxiety disorder was present in 46.8%, 42.9%, and 26.3% of the three groups respectively, and panic disorder in 53.3%, 44.1%, and 20.7% (both p < 0.001). Physical comorbidities were reported by 12.8%, 15.2%, and 7.6% across groups (p < 0.001), and psychiatric comorbidities by 4.4%, 5.6%, and 2.4% (p < 0.001).

Regarding occupational characteristics, seniority rank differed significantly across groups (p = 0.007). The mean working hours per week were 43.7 (SD = 25.2) for the definitive anginal pain

group, 44.7 (SD = 26.2) for possible anginal pain, and 42.3 (SD = 24.5) for those with no chest pain (p = 0.003). Country income level also differed significantly across groups (p < 0.001), with a higher proportion of participants from high-income countries in the never-experienced-chest-pain group (12.0%) compared to the definitive anginal pain group (9.7%).

Table 1. Sample Characteristics

Variables		Experienced definitive anginal pain (N=795)	Experienced possible anginal pain (N=2836)	Never experienced chest pain (N=2593)	Total (N=6224)	p value
Age, mean (SD)		29.3 (6.0)	29.1 (5.9)	29.1 (5.9)	29.1 (5.9)	0.797[1]
Sex (male)		352.0 (44.3%)	1533.0 (54.1%)	1564.0 (60.3%)	3449.0 (55.4%)	< 0.001[2]
Marital status	Single	493.0 (62.0%)	1845.0 (65.1%)	1726.0 (66.6%)	4064.0 (65.3%)	0.081[2]
	Married	288.0 (36.2%)	932.0 (32.9%)	833.0 (32.1%)	2053.0 (33.0%)	
	Separated	4.0 (0.5%)	23.0 (0.8%)	9.0 (0.3%)	36.0 (0.6%)	
	Divorced	10.0 (1.3%)	29.0 (1.0%)	20.0 (0.8%)	59.0 (0.9%)	
	Widowed	0.0 (0.0%)	7.0 (0.2%)	5.0 (0.2%)	12.0 (0.2%)	
Body mass index (kg/m^2), mean (SD)		25.9 (4.9)	25.9 (5.2)	25.5 (4.9)	25.7 (5.0)	0.016[1]
Body mass index categories	Healthy weight (18.5 - 24.9 kg/m^2)	329.0 (41.4%)	1233.0 (43.5%)	1214.0 (46.8%)	2776.0 (44.6%)	< 0.001[2]
	Underweight (<18.5 kg/m^2)	32.0 (4.0%)	100.0 (3.5%)	83.0 (3.2%)	215.0 (3.5%)	
	Overweight (25.0 - 29.9 kg/m^2)	307.0 (38.6%)	1005.0 (35.4%)	947.0 (36.5%)	2259.0 (36.3%)	
	Obesity (≥30.0 kg/m^2)	127.0 (16.0%)	498.0 (17.6%)	349.0 (13.5%)	974.0 (15.6%)	
Exercise frequency	None-exercising	458.0 (57.6%)	1507.0 (53.1%)	1393.0 (53.7%)	3358.0 (54.0%)	0.010[2]
	1-2 times/week	190.0 (23.9%)	703.0 (24.8%)	588.0 (22.7%)	1481.0 (23.8%)	
	3-5 times/week	119.0 (15.0%)	548.0 (19.3%)	538.0 (20.7%)	1205.0 (19.4%)	
	>5 times/week	28.0 (3.5%)	78.0 (2.8%)	74.0 (2.9%)	180.0 (2.9%)	
Physical comorbidities (yes)		102.0 (12.8%)	430.0 (15.2%)	196.0 (7.6%)	728.0 (11.7%)	< 0.001[2]
Psychiatric comorbidities (yes)		35.0 (4.4%)	158.0 (5.6%)	61.0 (2.4%)	254.0 (4.1%)	< 0.001[2]

Subjective sleep deprivation (yes)		440.0 (55.3%)	1417.0 (50.0%)	856.0 (33.0%)	2713.0 (43.6%)	< 0.001[2]
Anxiety disorder (yes)		372.0 (46.8%)	1218.0 (42.9%)	682.0 (26.3%)	2272.0 (36.5%)	< 0.001[2]
Panic disorder (yes)		424.0 (53.3%)	1252.0 (44.1%)	536.0 (20.7%)	2212.0 (35.5%)	< 0.001[2]
Nicotine product use	non-use	650.0 (81.8%)	2280.0 (80.4%)	2200.0 (84.8%)	5130.0 (82.4%)	< 0.001[2]
	Exclusive ends use	44.0 (5.5%)	184.0 (6.5%)	133.0 (5.1%)	361.0 (5.8%)	
	Exclusive tobacco use	83.0 (10.4%)	271.0 (9.6%)	206.0 (7.9%)	560.0 (9.0%)	
	Dual use	18.0 (2.3%)	101.0 (3.6%)	54.0 (2.1%)	173.0 (2.8%)	
Caffeinated drink consumption (yes)		724.0 (91.1%)	2548.0 (89.8%)	2330.0 (89.9%)	5602.0 (90.0%)	0.564[2]
Alcohol consumption (yes)		26.0 (3.3%)	109.0 (3.8%)	72.0 (2.8%)	207.0 (3.3%)	0.091[2]
Recreational drug use (yes)		35.0 (4.4%)	117.0 (4.1%)	86.0 (3.3%)	238.0 (3.8%)	0.198[2]
Academic/professional degree	Bachelor’s degree	613.0 (77.1%)	2246.0 (79.2%)	2081.0 (80.3%)	4940.0 (79.4%)	0.179[2]
	Diploma	23.0 (2.9%)	69.0 (2.4%)	66.0 (2.5%)	158.0 (2.5%)	
	Fellowship or board	47.0 (5.9%)	201.0 (7.1%)	150.0 (5.8%)	398.0 (6.4%)	
	Master’s degree	79.0 (9.9%)	219.0 (7.7%)	217.0 (8.4%)	515.0 (8.3%)	
	Doctorate degree	33.0 (4.2%)	101.0 (3.6%)	79.0 (3.0%)	213.0 (3.4%)	
Seniority	House officer	208.0 (26.2%)	678.0 (23.9%)	733.0 (28.3%)	1619.0 (26.0%)	0.007[2]
	General practitioner	230.0 (28.9%)	865.0 (30.5%)	770.0 (29.7%)	1865.0 (30.0%)	
	Resident	238.0 (29.9%)	923.0 (32.5%)	765.0 (29.5%)	1926.0 (30.9%)	
	Attending	119.0 (15.0%)	370.0 (13.0%)	325.0 (12.5%)	814.0 (13.1%)	
Years of experience, years		3.5 (5.0)	3.4 (4.9)	3.2 (4.6)	3.3 (4.8)	0.069[1]
Working hours/week, mean (SD)		43.7 (25.2)	44.7 (26.2)	42.3 (24.5)	43.6 (25.4)	0.003[1]
Working hours/week categories	<40 hours/week	339.0 (42.6%)	1170.0 (41.3%)	1118.0 (43.1%)	2627.0 (42.2%)	0.170[2]
	40-80 hours/week	401.0 (50.4%)	1448.0 (51.1%)	1318.0 (50.8%)	3167.0 (50.9%)	
	>80 hours/week	55.0 (6.9%)	218.0 (7.7%)	157.0 (6.1%)	430.0 (6.9%)	
Country income	Low	186.0 (23.4%)	668.0 (23.6%)	751.0 (29.0%)	1605.0 (25.8%)	< 0.001[2]
	Middle	532.0 (66.9%)	1957.0 (69.0%)	1531.0 (59.0%)	4020.0 (64.6%)	
	High	77.0 (9.7%)	211.0 (7.4%)	311.0 (12.0%)	599.0 (9.6%)	

[1]Linear Model ANOVA

[2]Pearson's Chi-squared test

Chest pain distribution varied significantly across medical specialties ($p = 0.005$) (Figure 1). Psychiatrists reported the highest prevalence of definitive anginal pain (21.7%), followed by Obstetrics & Gynaecology (18.9%) and Emergency Medicine (17.4%), while Neurosurgery had the lowest (4.8%).

Fig 1. Chest pain distribution by medical specialty.

Chest pain characteristics

The characteristics of chest pain among the 3,631 participants who experienced chest pain (definitive or possible anginal pain) are presented in Table 2. The most common pain sites in the definitive anginal pain group were the sternum upper middle (65.2%) and sternum lower (42.3%), whereas in the possible anginal pain group, the left anterior chest was the most commonly reported site (53.0%), followed by the sternum upper middle (32.2%). Chest pain had occurred more than three times in 54.3% and 55.3% of the definitive and possible anginal pain groups, respectively.

Emotion/excitement was the most frequently reported trigger in both groups (64.0% definitive; 59.9% possible), followed by coughing (34.3% vs. 22.0%) and cold wind (23.4% vs. 15.5%). Worsening with a chest cold or bad cough was reported by 54.3% of those with definitive anginal pain compared to 38.3% of those with possible anginal pain. In terms of pain quality, tightness and heaviness were more prevalent in the definitive anginal pain group, while stabbing pain was more common in the possible anginal pain group. The majority of participants

described their experience as discomfort rather than pain across both groups (68.7% and 62.3%, respectively).

Table 2. Chest Pain Characteristics

Variables		Experienced definitive anginal pain (N=795)	Experienced possible anginal pain (N=2836)	Total (N=3631)
Site	Left anterior chest	166.0 (20.9%)	1503.0 (53.0%)	1669.0 (46.0%)
	Left arm	61.0 (7.7%)	198.0 (7.0%)	259.0 (7.1%)
	Right chest	1.0 (0.1%)	56.0 (2.0%)	57.0 (1.6%)
	Sternum (lower)	336.0 (42.3%)	576.0 (20.3%)	912.0 (25.1%)
	Sternum (upper middle)	518.0 (65.2%)	912.0 (32.2%)	1430.0 (39.4%)
	All over chest	0.0 (0.0%)	49.0 (1.7%)	49.0 (1.3%)
	Others	3.0 (0.4%)	37.0 (1.3%)	40.0 (1.1%)
Experienced chest pain/discomfort >3 times		432.0 (54.3%)	1569.0 (55.3%)	2001.0 (55.1%)
Factors that induced chest pain/discomfort	Excitement or emotion	509.0 (64.0%)	1700.0 (59.9%)	2209.0 (60.8%)
	Stooping	46.0 (5.8%)	132.0 (4.7%)	178.0 (4.9%)
	Eating	92.0 (11.6%)	346.0 (12.2%)	438.0 (12.1%)
	Breathing	176.0 (22.1%)	513.0 (18.1%)	689.0 (19.0%)
	Cold wind	186.0 (23.4%)	440.0 (15.5%)	626.0 (17.2%)
	Coughing	273.0 (34.3%)	624.0 (22.0%)	897.0 (24.7%)
Chest pain gets worse with a chest cold or a bad cough		432.0 (54.3%)	1086.0 (38.3%)	1518.0 (41.8%)
General description of chest pain/discomfort	Pain	245.0 (30.8%)	1034.0 (36.5%)	1279.0 (35.2%)
	Discomfort	546.0 (68.7%)	1767.0 (62.3%)	2313.0 (63.7%)
	Both	2.0 (0.3%)	7.0 (0.2%)	9.0 (0.2%)
	Can't specify	2.0 (0.3%)	28.0 (1.0%)	30.0 (0.8%)
Specific description of the chest pain/discomfort	Burning	109.0 (13.7%)	359.0 (12.7%)	468.0 (12.9%)
	Pressure	286.0 (36.0%)	765.0 (27.0%)	1051.0 (28.9%)
	Stabbing	193.0 (24.3%)	876.0 (30.9%)	1069.0 (29.4%)
	Tightness	349.0 (43.9%)	1080.0 (38.1%)	1429.0 (39.4%)
	Heaviness	372.0 (46.8%)	878.0 (31.0%)	1250.0 (34.4%)

Factors associated with chest pain: Binary logistic regression

The crude and adjusted binary logistic regression analyses examining factors associated with combined chest pain (definitive and possible anginal pain vs. never experienced chest pain) are presented in Table 3. In the adjusted model, female sex was significantly associated with higher odds of chest pain (AOR = 1.49, 95% CI: 1.33–1.68, $p < 0.001$). Among BMI categories, obesity was the only category significantly associated with chest pain after adjustment (AOR = 1.30, 95% CI: 1.10–1.54, $p = 0.002$). Age was not significantly associated with chest pain in either the crude or adjusted analysis.

Physical comorbidities were significantly associated with increased odds of chest pain (AOR = 1.91, 95% CI: 1.59–2.30, $p < 0.001$). Among psychological factors, panic disorder showed the strongest association (AOR = 2.76, 95% CI: 2.44–3.13, $p < 0.001$), followed by sleep deprivation (AOR = 1.61, 95% CI: 1.43–1.80, $p < 0.001$) and anxiety disorder (AOR = 1.38, 95% CI: 1.23–1.56, $p < 0.001$).

All three nicotine product use categories were independently associated with chest pain after adjustment. Dual users had the highest odds (AOR = 1.53, 95% CI: 1.08–2.16, $p = 0.016$), followed by exclusive ENDS users (AOR = 1.33, 95% CI: 1.05–1.69, $p = 0.018$) and exclusive tobacco users (AOR = 1.29, 95% CI: 1.06–1.57, $p = 0.013$).

Regarding seniority rank, residents (AOR = 1.18, 95% CI: 1.01–1.37, $p = 0.042$) and attending physicians (AOR = 1.34, 95% CI: 1.03–1.76, $p = 0.032$) had significantly higher odds of chest pain compared to house officers. Country income level was also a significant predictor, with middle-income country participants showing higher odds of chest pain compared to high-income country participants (AOR = 1.71, 95% CI: 1.42–2.07, $p < 0.001$). Participants from low-income countries showed borderline significance (AOR = 1.23, 95% CI: 1.00–1.51, $p = 0.050$).

In a sensitivity analysis additionally including psychiatric comorbidities in the adjusted binary model, all primary predictors remained materially unchanged, with adjusted odds ratios within ±3% of the main model and preserved direction and statistical significance; psychiatric comorbidity was itself an independent predictor (AOR = 1.49, 95% CI: 1.09–2.04, p = 0.012).

Table 3. Factors Associated with Chest Pain (Combined Possible and Definite Anginal Chest Pain): Crude and Adjusted Analysis

Variable	OR (95% CI)	p-value	AOR* (95% CI)	p-value
Age (years)	1.00 (0.99–1.01)	0.71	0.99 (0.98–1.00)	0.11
Sex (reference: male)	1.41 (1.27–1.56)	<0.001	1.49 (1.33–1.68)	<0.001
Body mass index (reference: healthy weight)				
Underweight	1.24 (0.93–1.64)	0.144	1.31 (0.97–1.77)	0.084
Overweight	1.08 (0.96–1.20)	0.197	1.06 (0.93–1.20)	0.39
Obese	1.39 (1.20–1.62)	<0.001	1.30 (1.10–1.54)	0.002
Physical comorbidities (reference: no)	2.10 (1.77–2.49)	<0.001	1.91 (1.59–2.30)	<0.001
Sleep deprivation (reference: no)	2.12 (1.91–2.36)	<0.001	1.61 (1.43–1.80)	<0.001
Anxiety disorder (reference: no)	2.18 (1.96–2.43)	<0.001	1.38 (1.23–1.56)	<0.001
Panic disorder (reference: no)	3.29 (2.93–3.69)	<0.001	2.76 (2.44–3.13)	<0.001
Nicotine product use (reference: non-users)				
Exclusive ENDS users	1.29 (1.03–1.61)	0.025	1.33 (1.05–1.69)	0.018
Exclusive tobacco users	1.29 (1.08–1.55)	0.006	1.29 (1.06–1.57)	0.013
Dual users	1.65 (1.19–2.29)	0.002	1.53 (1.08–2.16)	0.016
Seniority (reference: house officer)				
General practitioner	1.18 (1.03–1.35)	0.018	1.13 (0.98–1.31)	0.104
Resident	1.26 (1.10–1.44)	<0.001	1.18 (1.01–1.37)	0.042
Attending	1.24 (1.05–1.48)	0.012	1.34 (1.03–1.76)	0.032
Country income level (reference: high)				
Low	1.23 (1.02–1.48)	0.032	1.23 (1.00–1.51)	0.05
Middle	1.76 (1.48–2.09)	<0.001	1.71 (1.42–2.07)	<0.001

Abbreviations: OR, odds ratio; AOR, adjusted odds ratio; 95% CI, 95% confidence interval; ENDS, electronic nicotine delivery system.

The reference is the never-experienced chest pain group

*Model fit: AIC = 7,704; McFadden's $R^2 = 0.0930$; AUC = 0.704; Sensitivity = 0.754; Specificity = 0.547; Accuracy = 0.667 (the cut-off value is set to 0.5)

Factors associated with chest pain: Multinomial logistic regression

To distinguish factors associated with definitive anginal pain from those associated with possible anginal pain, a multinomial logistic regression analysis was performed with never-experienced chest pain as the reference category. Results are presented in Table 4.

Female sex was significantly associated with both definitive anginal pain (AOR = 2.06, 95% CI: 1.72–2.47, $p < 0.001$) and possible anginal pain (AOR = 1.36, 95% CI: 1.21–1.54, $p < 0.001$). Among BMI categories, overweight (AOR = 1.22, 95% CI: 1.01–1.47, $p = 0.041$) and obese (AOR = 1.31, 95% CI: 1.02–1.69, $p = 0.036$) were significantly associated with definitive anginal pain, while only obesity was significant for possible anginal pain (AOR = 1.31, 95% CI: 1.11–1.56, $p = 0.002$). Age was not significantly associated with either outcome.

Physical comorbidities were significantly associated with both definitive anginal pain (AOR = 1.59, 95% CI: 1.22–2.07, $p < 0.001$) and possible anginal pain (AOR = 1.99, 95% CI: 1.65–2.40, $p < 0.001$). Panic disorder demonstrated the strongest association across both outcome categories (definitive: AOR = 3.52, 95% CI: 2.94–4.21; possible: AOR = 2.57, 95% CI: 2.26–2.93; both $p < 0.001$). Sleep deprivation was also significantly associated with both groups (definitive: AOR = 1.88, 95% CI: 1.58–2.23; possible: AOR = 1.56, 95% CI: 1.38–1.75; both $p < 0.001$), as was anxiety disorder (definitive: AOR = 1.38, 95% CI: 1.16–1.66; possible: AOR = 1.39, 95% CI: 1.22–1.57; both $p < 0.001$).

Regarding nicotine product use, exclusive tobacco users were significantly associated with definitive anginal pain (AOR = 1.54, 95% CI: 1.15–2.07, *p* = 0.004), while exclusive ENDS users did not reach significance for definitive anginal pain (AOR = 1.30, 95% CI: 0.90–1.88, *p* = 0.165). For possible anginal pain, all three nicotine use categories were significant: exclusive ENDS users (AOR = 1.35, 95% CI: 1.06–1.73, *p* = 0.016), exclusive tobacco users (AOR = 1.24, 95% CI: 1.00–1.52, *p* = 0.045), and dual users (AOR = 1.62, 95% CI: 1.14–2.30, *p* = 0.007).

Country income level was a significant predictor for possible anginal pain, with participants from middle-income countries having higher odds than those from high-income countries (AOR = 1.89, 95% CI: 1.55–2.30, *p* < 0.001), and participants from low-income countries also showing significantly higher odds (AOR = 1.36, 95% CI: 1.10–1.69, *p* = 0.005). For definitive anginal pain, only middle-income country participants showed a significant association (AOR = 1.46, 95% CI: 1.10–1.94, *p* = 0.009).

Table 4. Multinomial Logistic Regression Analysis of Factors Associated with Chest Pain

Variable	Definitive anginal pain				Possible anginal pain			
	**OR (95% CI)**	**p-value**	**AOR* (95% CI)**	**p-value**	**OR (95% CI)**	**p-value**	**AOR* (95% CI)**	**p-value**
Age	1.01 (0.99–1.02)	0.501	1.00 (0.99–1.02)	0.757	1.00 (0.99–1.01)	0.862	1.00 (0.99–1.01)	0.706
Sex (reference: male)	1.91 (1.63–2.25)	<0.001	2.06 (1.72–2.47)	<0.001	1.29 (1.16–1.44)	<0.001	1.36 (1.21–1.54)	<0.001
Body mass index (reference: healthy weight)								
Underweight	1.42 (0.93–2.18)	0.105	1.51 (0.96–2.35)	0.072	1.19 (0.88–1.60)	0.267	1.25 (0.91–1.72)	0.164
Overweight	1.20 (1.00–1.43)	0.048	1.22 (1.01–1.47)	0.041	1.05 (0.93–1.18)	0.469	1.02 (0.90–1.17)	0.725
Obese	1.34 (1.06–1.70)	0.015	1.31 (1.02–1.69)	0.036	1.41 (1.20–1.65)	<0.001	1.31 (1.11–1.56)	0.002
Physical comorbidities (reference: no)	1.80 (1.40–2.32)	<0.001	1.59 (1.22–2.07)	<0.001	2.19 (1.83–2.61)	<0.001	1.99 (1.65–2.40)	<0.001
Sleep deprivation (reference: no)	2.52 (2.14–2.96)	<0.001	1.88 (1.58–2.23)	<0.001	2.03 (1.82–2.26)	<0.001	1.56 (1.38–1.75)	<0.001

Anxiety disorder (reference: no)	2.46 (2.09–2.90)	<0.001	1.38 (1.16–1.66)	<0.001	2.11 (1.88–2.37)	<0.001	1.39 (1.22–1.57)	<0.001
Panic disorder (reference: no)	4.38 (3.70–5.19)	<0.001	3.52 (2.94–4.21)	<0.001	3.03 (2.69–3.42)	<0.001	2.57 (2.26–2.93)	<0.001
Nicotine product use (reference: non-users)								
Exclusive ENDS users	1.12 (0.79–1.59)	0.529	1.30 (0.90–1.88)	0.165	1.34 (1.06–1.68)	0.014	1.35 (1.06–1.73)	0.016
Exclusive tobacco users	1.36 (1.04–1.79)	0.024	1.54 (1.15–2.07)	0.004	1.27 (1.05–1.54)	0.014	1.24 (1.00–1.52)	0.045
Dual users	1.13 (0.66–1.94)	0.661	1.17 (0.67–2.04)	0.591	1.81 (1.29–2.52)	<0.001	1.62 (1.14–2.30)	0.007
Country income level (reference: high)								
Low	1.00 (0.74–1.35)	0.998	1.03 (0.76–1.42)	0.831	1.31 (1.07–1.61)	0.009	1.36 (1.10–1.69)	0.005
Middle	1.40 (1.07–1.84)	0.013	1.46 (1.10–1.94)	0.009	1.88 (1.56–2.27)	<0.001	1.89 (1.55–2.30)	<0.001

Abbreviations: OR, odds ratio; AOR, adjusted odds ratio; 95% CI, 95% confidence interval; ENDS, electronic nicotine delivery system.

The reference is the never-experienced chest pain group

*Model fit: AIC = 11,487; McFadden's R^2 = 0.0688

Discussion

This large multinational study examined the prevalence, characteristics, and determinants of chest pain among physicians across 12 MENA countries. Over half reported chest pain experience at least once (58.3%), with 12.8% meeting criteria for definitive anginal pain and 45.6% for possible anginal pain — substantially higher than estimates reported in general MENA region population studies (8,9). These figures are high for a relatively young working-age professional population and underscore the substantial burden of chest pain symptoms in this understudied occupational group. Collectively, the findings highlight the multifactorial nature of chest pain in this population, with psychological disorders—particularly panic and anxiety disorders—alongside sleep deprivation, physical comorbidities, nicotine product use, obesity, advancing seniority, and country income emerging as key determinants.

The prevalence of chest pain observed in this study substantially exceeds rates reported in general population surveys across the MENA region. National WHO STEPS surveys, which capture self-reported histories of heart attack, angina-type chest pain, and stroke as a composite endpoint, have reported prevalence estimates ranging from approximately 0.9% in Oman (30), to 6.1% in Kuwait (31), with intermediate estimates of 4.4% in Iraq (32), 3.3% in Morocco (33), 5.0% in the occupied Palestinian territory (34), and 4.7% in Lebanon (35). The considerably higher rates observed in our cohort — 12.8% definitive and 45.6% possible anginal pain — are not directly comparable to these general population estimates for several important methodological reasons. First, the STEPS composite endpoint conflates angina with prior myocardial infarction and stroke. Second, the RAQ, as used in this study, captures a broader spectrum of angina-like symptom experiences — including possible anginal pain — than clinical

diagnostic criteria or composite self-report items. Third, the occupational profile of our sample introduces systematic exposure to stressors — prolonged working hours, sleep deprivation, psychological comorbidity — that are independent risk factors for both cardiac and non-cardiac chest pain and are not present at comparable levels in general population cohorts. Taken together, these methodological distinctions suggest that the high chest pain burden observed in this cohort reflects the convergence of an elevated occupational risk environment with the inherent sensitivity of symptom-based classification, rather than indicating a higher prevalence of coronary artery disease per se. Nevertheless, even accounting for these differences, the magnitude of the prevalence observed — particularly for definitive anginal pain — remains clinically significant in a predominantly young professional population and reinforces the case for targeted occupational health surveillance in this group.

The pattern of symptom characteristics supports the validity of the classification approach used in this study. Definitive anginal pain showed a more classical characteristic pattern, with chest pain more frequently described as tightness and heaviness, whereas stabbing pain was more common among those with possible anginal pain. These differences are consistent with the established clinical distinction between more typical anginal pain and atypical or non-cardiac chest pain (36).

Female sex was one of the strongest and most consistent predictors of chest pain in our study, with a more pronounced association for definitive anginal pain than for possible anginal pain. This finding is noteworthy because chest pain, especially obstructive coronary artery disease, is often conceptualized as a predominantly male presentation (37,38). However, growing evidence suggests that women may experience ischemic symptoms through distinct mechanisms,

including coronary microvascular dysfunction, vasospasm, and non-obstructive coronary disease (38–40). Female healthcare workers may face compounded workplace stressors, including lower autonomy in hierarchical environments, gender-based discrimination, and the simultaneous burden of professional and domestic responsibilities. In the MENA context, these pressures may be further amplified by sociocultural expectations, potentially contributing to the observed sex difference (41,42).

The most significant finding of this study was the strong association between panic disorder and chest pain. Panic disorder emerged as the strongest predictor in the binary model and remained the strongest determinant in both definitive and possible anginal pain categories in the multinomial analysis. This finding is consistent with the established literature identifying panic disorder as a major cause of non-cardiac chest pain (6,43), but the magnitude of the association in this cohort is particularly notable. Chest pain is a frequent feature of panic attacks, and panic disorder is commonly identified among patients presenting with chest pain to emergency and outpatient settings (44,45). Interestingly, the association with panic disorder was stronger for definitive anginal pain than for possible anginal pain. Although this may seem counterintuitive, it is biologically plausible because panic attacks can produce intense substernal pressure, autonomic symptoms, and exertion-like symptom patterns that closely mimic classical angina (7). Moreover, panic disorder and cardiovascular disease are not mutually exclusive; chronic autonomic dysregulation, systemic inflammation, and hypothalamic-pituitary-adrenal (HPA) axis activation may increase long-term cardiovascular risk as well (14).

Anxiety disorder was also independently associated with chest pain in both models, with remarkably similar effect sizes across definitive and possible anginal pain. This pattern suggests

that anxiety may contribute to chest pain through a broader, non-specific mechanism, likely involving chronic sympathetic activation, somatic hypervigilance, heightened pain perception, and persistent muscle tension (5,46).

Sleep deprivation was another major factor, showing significant associations with chest pain, with a stronger association for definitive anginal pain. This pattern is clinically and biologically plausible. Sleep deprivation is known to increase sympathetic tone, blood pressure, systemic inflammation, endothelial dysfunction, and pain sensitivity. Among healthcare professionals, chronic sleep restriction is common because of shift work, on-call duties, and demanding schedules. The stronger association with definitive anginal pain raises the possibility that sleep disruption may not only intensify symptom perception but may also contribute to genuine cardiovascular stress and ischemia-like symptomatology (47,48).

Physical comorbidities were independently associated with chest pain, with a stronger association for possible anginal pain than for definitive anginal pain. This likely reflects the contribution of non-cardiac conditions such as gastroesophageal reflux disease, respiratory disease, and musculoskeletal disorders, all of which can present with recurrent chest pain (4). At the same time, some physical comorbidities—particularly hypertension and diabetes—may also contribute to true cardiovascular risk (49). In a relatively young cohort, however, the overall pattern likely reflects a mixture of both cardiac and non-cardiac mechanisms.

Obesity also emerged as an important determinant. It was the only BMI category that remained significantly associated with chest pain. This is consistent with the multifaceted effects of obesity on chest pain, including increased cardiac workload, gastroesophageal reflux, sleep-disordered breathing, reduced exercise tolerance, and musculoskeletal strain (50). The near-identical

associations across both subtypes suggest that obesity contributes to chest pain through overlapping cardiac and non-cardiac pathways. The additional finding that overweight status was associated with definitive anginal pain but not possible anginal pain may indicate a gradient effect, whereby increasing adiposity is more strongly linked to the more typical anginal phenotype (51).

Nicotine product use showed important subtype-specific patterns. Conventional tobacco use was associated with both definitive and possible anginal pain, with a stronger effect for definitive anginal pain. This is fully consistent with the established cardiotoxic effects of tobacco, including endothelial dysfunction, platelet activation, inflammation, and coronary vasospasm, which can directly contribute to more classical ischemic pain patterns. In contrast, exclusive ENDS use was associated with possible anginal pain but not definitive anginal pain. This distinction is important because it suggests that ENDS-related chest symptoms in this population may be more likely driven by non-cardiac mechanisms such as airway irritation, respiratory inflammation, or nicotine-induced sympathomimetic effects rather than overt myocardial ischemia (52,53). Dual use was significantly associated with possible anginal pain but not definitive anginal pain, although this may partly reflect limited statistical power because of the relatively small number of dual users. Another possibility is that dual users may have somewhat different cumulative smoke exposure profiles than exclusive tobacco users (53).

Seniority also showed an important association. Residents and attending physicians had higher odds of chest pain than house officers in the adjusted binary model, suggesting a gradient consistent with cumulative occupational stress and allostatic load. More senior physicians often carry greater clinical responsibility, administrative burden, and medicolegal pressure, all of

which may amplify stress-related symptom burden (11,54). Although seniority was not retained in the multinomial model, its significance in the binary model suggests that it may predispose more broadly to chest pain in general rather than to a specific anginal subtype. Working hours, despite showing significance in univariate analyses, did not remain significant in adjusted models, indicating that their effect may be mediated through downstream variables such as sleep deprivation, anxiety, and physical health.

Country income level was another notable determinant. Participants from middle-income countries had higher odds of chest pain relative to those from high-income countries, both in binary and multinomial analyses, while low-income country status was particularly associated with possible anginal pain. These findings likely reflect structural differences in healthcare systems, occupational support, staffing ratios, workload intensity, remuneration, and access to preventive or mental health services (9,15). The stronger association with possible anginal pain may suggest that psychosocial stressors and untreated non-cardiac conditions contribute substantially to chest pain burden in lower-resource settings (55,56).

Taken together, these findings have several practical implications for occupational health policy and clinical practice. First, the very high prevalence of chest pain supports the need for systematic screening programmes within healthcare institutions that address both cardiovascular and psychological health. Second, the dominant role of panic disorder, anxiety, and sleep deprivation suggests that mental health support and sleep health interventions should be central—not secondary—components of occupational health programmes. Third, the distinct associations of tobacco, ENDS, and dual use with chest pain subtypes indicate that all nicotine products should be targeted in cessation counselling, rather than focusing solely on conventional

tobacco. Finally, the associations with seniority and country income point to the need for institution-level and policy-level strategies, particularly for senior clinicians and those working in lower-resource health systems.

Strengths and limitations

This study has several strengths. It represents one of the largest multinational investigations of chest pain among physicians, including 6,224 physicians from 12 countries in the MENA region, providing substantial statistical power and enhancing the robustness of the findings. The use of standardized instruments to assess chest pain, anxiety, and panic disorders, along with the application of multivariable regression models, strengthens the internal validity of the results.

However, several limitations should be acknowledged. The cross-sectional design precludes conclusions about temporality or causality. Chest pain classification was based on the self-reported RAQ rather than clinical evaluation, electrocardiography, or biomarkers, and therefore cannot definitively distinguish ischemic from non-ischemic causes. This limitation is particularly salient given the high prevalence of panic disorder in our cohort: panic attacks can be precipitated by exertion and subside with rest, mimicking the exact RAQ criteria for definitive anginal pain. This overlap may partly explain the stronger association of panic disorder with definitive than possible anginal pain and suggests RAQ specificity is attenuated in populations with high panic disorder prevalence. The very high prevalence of possible anginal pain likely includes a heterogeneous mix of gastrointestinal, musculoskeletal, respiratory, and psychogenic etiologies. All exposures were self-reported, introducing the possibility of recall bias and social desirability bias, particularly for psychological disorders and substance use. Although the sample was large and multinational, online recruitment may have introduced selection bias toward more

engaged or digitally connected physicians. As Egypt, Iraq, and Yemen together contributed 56.0% of the sample, the country-income findings largely reflect these three settings rather than the MENA region as a whole, and country-level analysis was precluded for underrepresented countries. Finally, residual confounding from unmeasured variables—such as family history, dietary patterns, or specific occupational exposures—cannot be excluded.

Conclusion

Chest pain was highly prevalent among physicians in this large MENA-region cohort, with the pattern of determinants suggesting that psychological, occupational, and behavioral factors — particularly panic disorder, anxiety, sleep deprivation, obesity, and nicotine product use — are major drivers alongside traditional cardiovascular risk factors. While the symptom profiles and risk factor patterns are consistent with a substantial non-cardiac contribution in this relatively young professional population, the prevalence of definitive anginal pain and the associations with tobacco use and obesity indicate that true cardiovascular pathology cannot be discounted. These findings call for an integrated occupational health response that addresses both psychological and cardiovascular risk, incorporating mental health support, sleep health interventions, and nicotine cessation alongside cardiac surveillance. Longitudinal studies with objective clinical endpoints are needed to better characterize the long-term cardiovascular consequences of this symptom burden.

Acknowledgment

We acknowledge the substantial role of the Dijla Academic Research Students (DARS) Consortium in data collection for this study, and the Medical Research Group of Egypt (Negida Academy, Arlington, Massachusetts, USA) for their support during the study. We want to extend our appreciation to Dr. Mouhammed Tarek Hasan for critically revising the manuscript**.**

Author contributions:

SMS contributed to the study's design, methodology, validation, sample size calculation, survey design, data curation, conducting the formal analysis, interpreting the results, drafting the manuscript, and critically revising the manuscript. WA contributed to the interpretation of the results and drafting of the manuscript. AA was involved in data coordination and reviewing the manuscript. KG contributed to conceptualization and data curation. OH was involved in the survey design and data curation. MA was involved in data coordination and reviewing the manuscript. OA, SA, NO, HA, and BMR contributed to the data collection and reviewing of the manuscript. RSE revised the protocol and critically revised the manuscript. The DARS consortium members were responsible for data collection.

Dijla Academic Research Students (DARS) Consortium

Mohammed Tarek Hasan (Faculty of Medicine, Al-Azhar University, Cairo, Egypt, mohammedtarekhasan1@outlook.com) critically revised the manuscript.

Dijla Academic Research Students (DARS) consortium:

Ahmed Farid (ORCID ID: 0009-0006-2787-0024) served as a data collector.

Ali Algawoog served as a data collector.

Ali Tirihbat (ORCID ID: 0000-0003-2852-4829) served as a data collector.

Angham Hassanin, ORCID ID: 0009-0000-6858-1305) served as a data collector.

Areen Harahsheh (ORCID ID: 0009-0001-8741-7460, served as a data collector.

Dhurgham Alomar (ORCID ID: 0000-0002-9069-0249) served as a data collector.

Farah Najim Abdulzahra (ORCID ID: 0009-0005-4567-701X) served as a data collector.

Khaled Matter (ORCID ID: 0009-0004-9512-7959) served as a data collector.

Linda Alqassass served as a data collector.

Makia Mohamed (ORCID ID: 0009-0002-2905-4961) served as a data collector.

Mina Faragalla served as a data collector.

Mohamed Elmatbouly (ORCID ID: 0000-0002-0257-9656) served as a data collector.

Mohammed Sarhan Alkhallatee (ORCID ID: 0009-0003-9439-5129) served as a data collector.

Mostafa Abdelraheem served as a data collector.

Nermeen Hamdi served as a data collector.

Radfan Al-Abdi (ORCID ID: 0000-0002-1741-1397) served as a data collector.

Ragda Mamoun (ORCID ID: 0009-0006-7864-6209) served as a data collector.

Safa Awata served as a data collector.

Saif Aldein Afify served as a data collector.

Samiha Al-Hubaishi (ORCID ID: 0009-0007-5709-1379) served as a data collector.

Tala Iyad Alanati (ORCID ID: 0009-0005-3688-4586) served as a data collector.

Toqa Mohamed (ORCID ID: 0009-0009-6256-1392) served as a data collector.

Conflict of interest

We have no conflict of interest to disclose.

Funding

No funding was received for conducting this study.

Data availability

The data underlying this article will be shared on reasonable request to the corresponding author.

Supporting files

S1. Supplementary Table 1: Sample distribution.

S2. Supplementary Table 2: STROBE Statement—Checklist of items that should be included in reports of cross-sectional studies

S3. Supplementary Table 3. Univariate binomial regression analysis for individual variables with chest pain (combined possible and definite anginal chest pain) as the dependent variable, and never experienced chest pain as the reference.

S4. Supplementary Table 4. Univariate multinomial regression analysis for individual variables with chest pain (possible vs definite anginal chest pain) as the dependent variable, and never experienced chest pain as the reference

References

1. Dawson LP, Andrew E, Nehme Z, Bloom J, Okyere D, Cox S, et al. Incidence, diagnoses and outcomes of ambulance attendances for chest pain: a population-based cohort study. Ann Epidemiol. 2022 Aug;72:32–9.

2. Dawson LP, Smith K, Cullen L, Nehme Z, Lefkovits J, Taylor AJ, et al. Care Models for Acute Chest Pain That Improve Outcomes and Efficiency: JACC State-of-the-Art Review. J Am Coll Cardiol. 2022 Jun 14;79(23):2333–48.

3. Fass R, Achem SR. Noncardiac chest pain: epidemiology, natural course and pathogenesis. J Neurogastroenterol Motil. 2011 Apr 27;17(2):110–23.

4. Campbell KA, Madva EN, Villegas AC, Beale EE, Beach SR, Wasfy JH, et al. Non-cardiac Chest Pain: A Review for the Consultation-Liaison Psychiatrist. Psychosomatics. 2017;58(3):252–65.

5. Gonzalez-Ibarra F, Cruz-Ruiz M, Murillo Llanes J, Achem SR, Fass R. The role of psychological factors in noncardiac chest pain of esophageal origin. J Neurogastroenterol Motil. 2024 Jul 30;30(3):272–80.

6. Soares-Filho GLF, Arias-Carrión O, Santulli G, Silva AC, Machado S, Valenca AM, et al. Chest pain, panic disorder and coronary artery disease: a systematic review. CNS Neurol Disord Drug Targets. 2014;13(6):992–1001.

7. Huffman JC, Pollack MH, Stern TA. Panic disorder and chest pain: mechanisms, morbidity, and management. Prim Care Companion J Clin Psychiatry. 2002 Apr;4(2):54–62.

8. Soleimani H, Nasrollahizadeh A, Nasrollahizadeh A, Razeghian I, Molaei MM, Hakim D, et al. Cardiovascular disease burden in the North Africa and Middle East region: an analysis of the global burden of disease study 1990-2021. BMC Cardiovasc Disord. 2024 Dec 19;24(1):712.

9. Manla Y, Almahmeed W. The pandemic of coronary heart disease in the middle east and north africa: what clinicians need to know. Curr Atheroscler Rep. 2023 Sep;25(9):543–57.

10. Alhajaji R, Alfahmi MZ, Alshaikhi SA, Fairaq AM, Fudlaldeen Jan S, Aljuaid S, et al. The influence of workplace stressors on the risk of cardiovascular diseases among healthcare providers: a systematic review. Front Psychiatry. 2025 Aug 21;16:1461698.

11. Shanafelt TD, Hasan O, Dyrbye LN, Sinsky C, Satele D, Sloan J, et al. Changes in burnout and satisfaction with work-life balance in physicians and the general US working population between 2011 and 2014. Mayo Clin Proc. 2015 Dec;90(12):1600–13.

12. Kivimäki M, Leino-Arjas P, Luukkonen R, Riihimäki H, Vahtera J, Kirjonen J. Work stress and risk of cardiovascular mortality: prospective cohort study of industrial employees. BMJ. 2002 Oct 19;325(7369):857.

13. Chemali Z, Ezzeddine FL, Gelaye B, Dossett ML, Salameh J, Bizri M, et al. Burnout among healthcare providers in the complex environment of the Middle East: a systematic review. BMC Public Health. 2019 Oct 22;19(1):1337.

14. Tully PJ, Turnbull DA, Beltrame J, Horowitz J, Cosh S, Baumeister H, et al. Panic disorder and incident coronary heart disease: a systematic review and meta-regression in 1131612 persons and 58111 cardiac events. Psychol Med. 2015 Oct;45(14):2909–20.

15. Mokdad AH, Forouzanfar MH, Daoud F, El Bcheraoui C, Moradi-Lakeh M, Khalil I, et al. Health in times of uncertainty in the eastern Mediterranean region, 1990-2013: a systematic analysis for the Global Burden of Disease Study 2013. Lancet Glob Health. 2016 Oct;4(10):e704-13.

16. Elbarazi I, Loney T, Yousef S, Elias A. Prevalence of and factors associated with burnout among health care professionals in Arab countries: a systematic review. BMC Health Serv Res. 2017 Jul 17;17(1):491.

17. Alhassan MA, Alarabi MA, Alhassan SA, Albalawi WM, AlRabiah ES. Prevalence of burnout and its risk and protective factors among healthcare workers in the Middle East, North Africa, and Turkey: a systematic review and meta-analysis. Front Psychol. 2025 Oct 22;16:1539105.

18. von Elm E, Altman DG, Egger M, Pocock SJ, Gøtzsche PC, Vandenbroucke JP, et al. The Strengthening the Reporting of Observational Studies in Epidemiology (STROBE) statement: guidelines for reporting observational studies. Ann Intern Med. 2007 Oct 16;147(8):573–7.

19. World Medical Association. World Medical Association Declaration of Helsinki: ethical principles for medical research involving human subjects. JAMA. 2013 Nov 27;310(20):2191–4.

20. World Bank Country and Lending Groups – World Bank Data Help Desk [Internet]. [cited 2026 Apr 16]. Available from: https://datahelpdesk.worldbank.org/knowledgebase/articles/906519-world-bank-country-and-lending-groups

21. World Bank Group country classifications by income level for FY24 (July 1, 2023- June 30, 2024) [Internet]. [cited 2026 Apr 16]. Available from: https://blogs.worldbank.org/en/opendata/new-world-bank-group-country-classifications-income-level-fy24

22. Rose GA. The diagnosis of ischaemic heart pain and intermittent claudication in field surveys. Bull World Health Organ. 1962;27:645–58.

23. Fischbacher CM, Bhopal R, Unwin N, White M, Alberti KG. The performance of the Rose angina questionnaire in South Asian and European origin populations: a comparative study in Newcastle, UK. Int J Epidemiol. 2001 Oct;30(5):1009–16.

24. Rahman MA, Spurrier N, Mahmood MA, Rahman M, Choudhury SR, Leeder S. Rose Angina Questionnaire: validation with cardiologists' diagnoses to detect coronary heart disease in Bangladesh. Indian Heart J. 2013 Feb;65(1):30–9.

25. Spitzer RL, Kroenke K, Williams JBW, Löwe B. A brief measure for assessing generalized anxiety disorder: The GAD-7. Arch Intern Med. 2006 May 22;166(10):1092–7.

26. Batterham PJ, Mackinnon AJ, Christensen H. The panic disorder screener (PADIS): Development of an accurate and brief population screening tool. Psychiatry Res. 2015 Jul 30;228(1):72–6.

27. Naing L, Nordin RB, Abdul Rahman H, Naing YT. Sample size calculation for prevalence studies using Scalex and ScalaR calculators. BMC Med Res Methodol. 2022 Jul 30;22(1):209.

28. Wang M, Ding Q, Sang L, Song L. Prevalence of Pain and Its Risk Factors Among ICU Personnel in Tertiary Hospital in China: A Cross-Sectional Study. J Pain Res. 2022 Jun 20;15:1749–58.

29. Serdar CC, Cihan M, Yücel D, Serdar MA. Sample size, power and effect size revisited: simplified and practical approaches in pre-clinical, clinical and laboratory studies. Biochem Med (Zagreb). 2021 Feb 15;31(1):010502.

30. World Health Organization. Oman World Health Survey: Oman STEPS Survey 2017 – data book. Geneva: World Health Organization; 2017 [cited 2026 Apr 15]. Available from: https://cdn.who.int/media/docs/default-source/ncds/ncd-surveillance/data-reporting/oman/steps/oman-steps-2017-data-book.pdf

31. World Health Organization. Kuwait STEPS Survey 2014: Kuwait STEPS noncommunicable diseases risk factors survey 2014. Geneva: World Health Organization; 2015 [cited 2026 Apr 15]. Available from: https://cdn.who.int/media/docs/default-source/ncds/ncd-surveillance/data-reporting/kuwait/steps/kuwait-2014-steps-report.pdf

32. World Health Organization. Iraq National STEPwise Survey for noncommunicable diseases risk factors 2015. Geneva: World Health Organization; 2018 [cited 2026 Apr 15]. Available from: https://extranet.who.int/fctcapps/sites/default/files/2023-04/iraq_2018_annex-1_STEPS_report_2015.pdf

33. World Health Organization. Morocco STEPS Survey 2017–2018: final report. Geneva: World Health Organization; 2018 [cited 2026 Apr 15]. Available from: https://cdn.who.int/media/docs/default-source/ncds/ncd-surveillance/data-reporting/morocco/steps/steps-report-2017-2018-morocco-final.pdf

34. World Health Organization. National STEPwise Survey for noncommunicable diseases risk factors in the occupied Palestinian territory 2022. Geneva: World Health Organization; 2023 [cited 2026 Apr 15]. Available from: https://cdn.who.int/media/docs/default-source/2021-dha-docs/steps_2022_reportfinal.pdf

35. World Health Organization. Lebanon STEPS Survey 2016–2017. Geneva: World Health Organization; 2018 [cited 2026 Apr 15]. Available from: https://cdn.who.int/media/docs/default-source/ncds/ncd-surveillance/data-reporting/lebanon/steps/lebanon-steps-report-2016-2017.pdf

36. Collet J-P, Thiele H, Barbato E, Barthélémy O, Bauersachs J, Bhatt DL, et al. 2020 ESC Guidelines for the management of acute coronary syndromes in patients presenting without persistent ST-segment elevation. Eur Heart J. 2021 Apr 7;42(14):1289–367.

37. Bugiardini R, Bairey Merz CN. Angina with “normal” coronary arteries: a changing philosophy. JAMA. 2005 Jan 26;293(4):477–84.

38. Maas A, Cenko E, Vaccarino V, Göttgens I, Bergami M, Manfrini O, et al. Changing clinical perspectives on sex and healthcare disparities in ischaemic heart disease. Lancet Reg Health Eur. 2025 Sep;56:101370.

39. Reynolds HR, Bairey Merz CN, Berry C, Samuel R, Saw J, Smilowitz NR, et al. Coronary arterial function and disease in women with no obstructive coronary arteries. Circ Res. 2022 Feb 18;130(4):529–51.

40. Mehta PK, Huang J, Levit RD, Malas W, Waheed N, Bairey Merz CN. Ischemia and no obstructive coronary arteries (INOCA): A narrative review. Atherosclerosis. 2022 Dec;363:8–21.

41. Al-Dossary RN. Leadership style, work engagement and organizational commitment among nurses in saudi arabian hospitals. J Healthc Leadersh. 2022 Jun 7;14:71–81.

42. Abuhammad S, Alzoubi KH, Al-Azzam S, Karasneh R, Masadeh MM, Alameddine M. Work-life balance, job satisfaction, and burnout among nurses in Jordan: A cross-sectional study. PLoS ONE. 2025 Oct 16;20(10):e0334603.

43. Katerndahl DA, Trammell C. Prevalence and recognition of panic states in STARNET patients presenting with chest pain. J Fam Pract. 1997 Jul;45(1):54–63.

44. Fleet RP, Dupuis G, Marchand A, Burelle D, Arsenault A, Beitman BD. Panic disorder in emergency department chest pain patients: prevalence, comorbidity, suicidal ideation, and physician recognition. Am J Med. 1996 Oct;101(4):371–80.

45. Huffman JC, Pollack MH. Predicting panic disorder among patients with chest pain: an analysis of the literature. Psychosomatics. 2003;44(3):222–36.

46. White KS, Craft JM, Gervino EV. Anxiety and hypervigilance to cardiopulmonary sensations in non-cardiac chest pain patients with and without psychiatric disorders. Behav Res Ther. 2010 May;48(5):394–401.

47. Holmer BJ, Lapierre SS, Jake-Schoffman DE, Christou DD. Effects of sleep deprivation on endothelial function in adult humans: a systematic review. Geroscience. 2021 Feb 9;43(1):137–58.

48. Kohansieh M, Makaryus AN. Sleep deficiency and deprivation leading to cardiovascular disease. Int J Hypertens. 2015 Oct 1;2015:615681.

49. Visseren FLJ, Mach F, Smulders YM, Carballo D, Koskinas KC, Bäck M, et al. 2021 ESC Guidelines on cardiovascular disease prevention in clinical practice. Eur Heart J. 2021 Sep 7;42(34):3227–337.

50. Poirier P, Giles TD, Bray GA, Hong Y, Stern JS, Pi-Sunyer FX, et al. Obesity and cardiovascular disease: pathophysiology, evaluation, and effect of weight loss. Arterioscler Thromb Vasc Biol. 2006 May;26(5):968–76.

51. Lavie CJ, Milani RV, Ventura HO. Obesity and cardiovascular disease: risk factor, paradox, and impact of weight loss. J Am Coll Cardiol. 2009 May 26;53(21):1925–32.

52. Park J-A, Crotty Alexander LE, Christiani DC. Vaping and lung inflammation and injury. Annu Rev Physiol. 2022 Feb 10;84:611–29.

53. Behrooz L, Xie W, Goghari A, Robertson R, Bhatnagar A, Stokes A, et al. Electronic cigarette use and chest pain in US adults: Evidence from the PATH study. Tob Induc Dis. 2024 Jan 19;22.

54. Dyrbye LN, Thomas MR, Power DV, Durning S, Moutier C, Massie FS, et al. Burnout and serious thoughts of dropping out of medical school: a multi-institutional study. Acad Med. 2010 Jan;85(1):94–102.

55. Patel V, Kleinman A. Poverty and common mental disorders in developing countries. Bull World Health Organ. 2003 Oct 14;81(8):609–15.

56. Vedanthan R, Fuster V. Cardiovascular disease in Sub-Saharan Africa: a complex picture demanding a multifaceted response. Nat Clin Pract Cardiovasc Med. 2008 Sep;5(9):516–7.

Figure

Click here to access/download;Figure;Figure 1.tiff

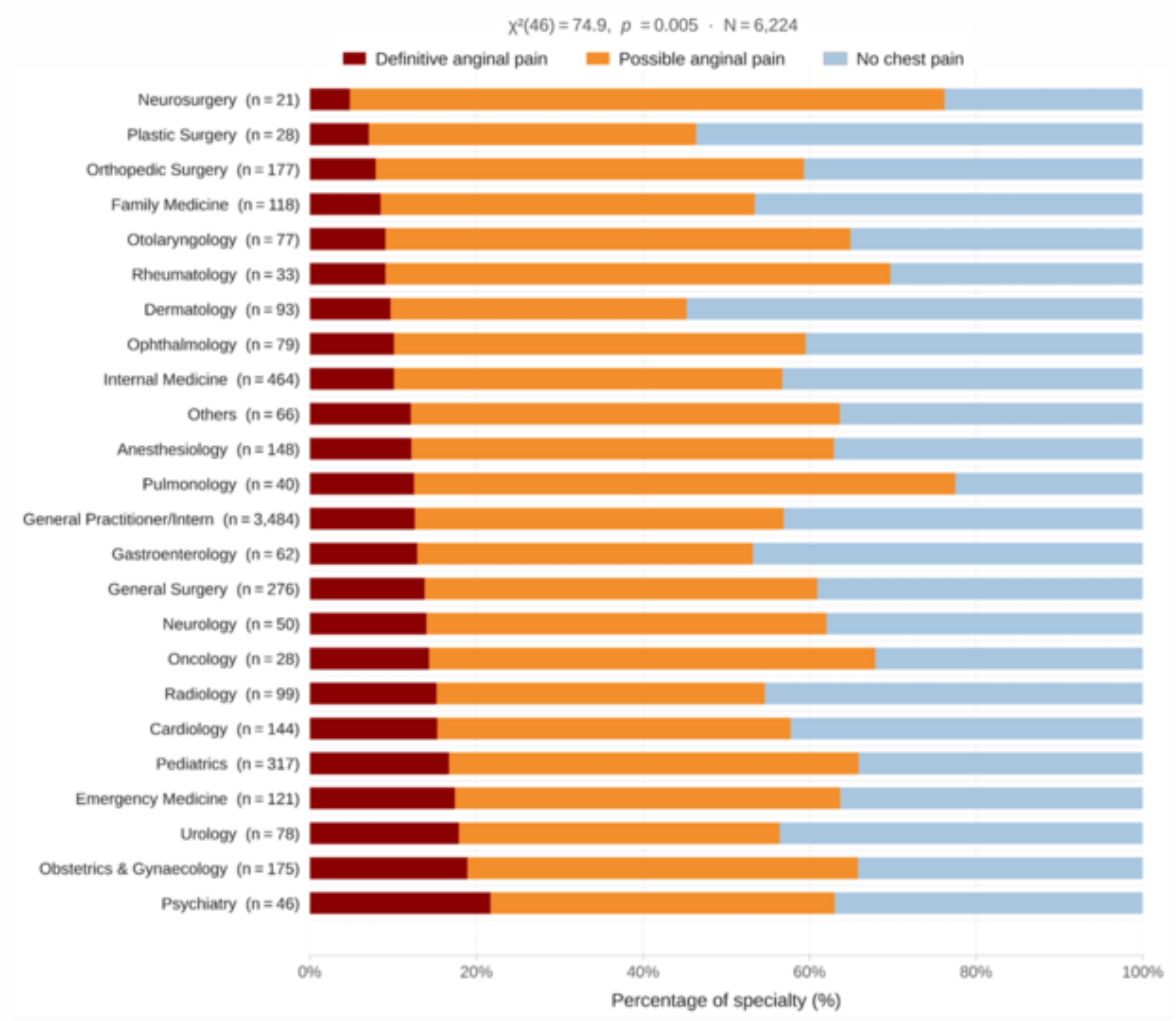

Click here to access/download
Supporting Information
Supplementary Table 1 (1).docx

Click here to access/download
Supporting Information
Supplementary Table 2 Plos one.docx

Click here to access/download
Supporting Information
Supplementary Table 3 (1).docx

Click here to access/download
Supporting Information
Supplementary Table 4 (1).docx

Dear in Plos one

Thank you for your message regarding the ethics approval documentation. As requested, we have already sent the English version. Unfortunately, due to the ongoing conflict in Yemen, we have the attached copy only .

Could you kindly confirm whether the documents we have provided are sufficient for your review, or advise if there are any specific additional materials you require from us? At present, the approvals already submitted represent the full extent of what is available to our team.

We appreciate your understanding of the challenges faced in securing documentation from conflict-affected regions and remain committed to ensuring compliance with your requirements.

Sincerely,

Weam Aldiban
