# Supplementary material for: Prevalence, characteristics, and determinants of chest pain among physicians in the Middle East and North Africa: A multinational cross-sectional study
Source: PLoS One. 2026 Aug 31;21(8):e0355946. doi: 10.1371/journal.pone.0355946 (PMC13529011; doi:10.1371/journal.pone.0355946)
Supplement: Supplementary file 7 [file pone.0355946.s009.docx]

Dear in Plos one

Thank you for your message regarding the ethics approval documentation. As requested, we have already sent the English version. Unfortunately, due to the ongoing conflict in Yemen, we have the attached copy only .

Could you kindly confirm whether the documents we have provided are sufficient for your review, or advise if there are any specific additional materials you require from us? At present, the approvals already submitted represent the full extent of what is available to our team.

We appreciate your understanding of the challenges faced in securing documentation from conflict-affected regions and remain committed to ensuring compliance with your requirements.

Sincerely,

Weam Aldiban
