# Supplementary material for: Prevalence, characteristics, and determinants of chest pain among physicians in the Middle East and North Africa: A multinational cross-sectional study
Source: PLoS One. 2026 Aug 31;21(8):e0355946. doi: 10.1371/journal.pone.0355946 (PMC13529011; doi:10.1371/journal.pone.0355946)
Supplement: Supplementary file 8 [file pone.0355946.s010.docx]

**Response to Reviewers**

**Manuscript PONE-D-26-19587**

*Prevalence, characteristics, and determinants of chest pain among physicians in the Middle East and North Africa: A multinational cross-sectional study*

Dear Dr. Shrestha and Reviewers,

We thank the Academic Editor and both reviewers for their careful and constructive evaluation of our manuscript. The comments have substantially improved the work. Below we respond to each point individually. Reviewer comments are shown in italics with a coloured rule; our responses follow.

# Responses to the Academic Editor / Journal Requirements

## 1. Style and file-naming requirements

***Editor:*** *Please ensure the manuscript meets PLOS ONE style requirements, including file naming, using the PLOS ONE templates.*

**Response:** We have reformatted the title page, main body, and supporting files in accordance with the PLOS ONE formatting templates and have renamed all files to conform to the journal's file-naming conventions*.*

## 2. Inclusivity in Global Research questionnaire

***Editor:*** *Please include a completed copy of PLOS' questionnaire on inclusivity in global research.*

**Response:** We have completed the PLOS Inclusivity in Global Research questionnaire and uploaded it as Supporting Information with the revised submission.

## 3. Identifying data in Supporting Information

***Editor:*** *Identifying data were present in a Supporting Information file, which has been removed. Personally identifying data must not be shared; please remove or anonymise and re-upload a fully anonymised dataset (columns deleted, not hidden).*

**Response:** We understand that the file flagged is Supplementary Table 1 (Sample distribution). We respectfully clarify that this file contains only the aggregate distribution of participants across the 12 participating countries: it comprises summary counts, not individual-level records, and includes no names, identifiers, dates, contact details, or other personally identifying fields. We therefore submit that it does not constitute identifying participant data and would be grateful if it could be reinstated; should the editorial office nonetheless prefer its removal or modification, we are happy to comply. Separately, for the individual-level data underlying our analyses, we now provide a fully de-identified minimal dataset as Supporting Information (see Reviewer 2, point 1), with all directly and indirectly identifying variables deleted (not hidden).

## 4. Reviewer-recommended citations

***Editor:*** *If reviewers recommend specific citations, evaluate their relevance; there is no obligation to cite unless directed.*

**Response:** We have evaluated each reference implied by the reviewers' comments and incorporated those that strengthen the manuscript, as detailed in the relevant responses below.

# Responses to Reviewer #1

**Response:** We thank Reviewer #1 for the detailed annotations on the manuscript PDF and for recognising the importance and scale of the study. We address the general comments first, followed by each in-text annotation.

## General comments

***Reviewer 1 (G1):*** *A few countries have disproportionately large numbers of respondents, so it may not be appropriate to generalise to MENA countries.*

**Response:** We thank the reviewer and Reviewer #2 for noting the country distribution. We respectfully clarify that we did not present the findings as representative of every MENA country: the sampling and its uneven country distribution are already acknowledged as a limitation in the original manuscript. We note that the great majority of published studies on the MENA region likewise do not encompass all MENA countries, and that a sample spanning 12 MENA countries is reasonably described as a MENA-region study. To address the reviewer’s concern directly, we have nonetheless strengthened the existing limitation to quantify the clustering (Egypt, Iraq, Yemen, and Jordan contributed 67.3% of the sample; Egypt alone 29.0%) and to caution explicitly against country-level generalisation to under-represented settings **(Page: 31; Lines: 519-520)**.

***Reviewer 1 (G2):*** *Aims and objectives and the methodology should be very clear. The study-specific proforma/questionnaire is not clear.*

**Response:** We respectfully note that the Introduction already concludes with an explicit aim (“Therefore, this study aimed to determine the prevalence, clinical characteristics, and determinants of chest pain among physicians in the MENA region”), and that, per the STROBE guidance under which this manuscript is reported, study objectives belong in the Introduction rather than the Methods. To address the reviewer’s concern, we have lightly expanded this closing sentence to distinguish the primary objective (estimating the prevalence of chest pain) from the secondary objectives (describing its characteristics and identifying its determinants) **(Page: 7; Lines: 111-113)**. Regarding the study instrument, the standardized components (Rose Angina Questionnaire, GAD-7, and PADIS) are validated and fully cited; the remaining items were single, author-developed direct questions (e.g., self-reported exercise frequency, sleep deprivation, and comorbidities). To make the instrument fully transparent, we have compiled these author-developed items, with their exact wording, into a one-page Supporting Information file, now referenced in the Methods (see annotation 5).

***Reviewer 1 (G3):*** *Some language/grammar policing is needed.*

**Response:** The manuscript has undergone a thorough language and grammar revision by a fluent English-proficient author.

## In-text annotations

***Annotation 1 (L62, “anxiety disorder”):*** *The term “anxiety disorder” is potentially misleading here. The GAD-7 is a screening tool for anxiety symptoms or probable generalized anxiety disorder. If you use the term 'anxiety disorder' here it could be interpreted as including both GAD and panic disorder.*

***Annotation 11 (L284):*** *The GAD-7 is a screening instrument and does not diagnose an anxiety disorder. It is used to assess the severity of anxiety symptoms or to identify probable generalized anxiety disorder. Please revise the wording accordingly.*

**Response:** We thank the reviewer for this important point and agree that the GAD-7 and PADIS are screening instruments that quantify symptom severity and do not, by themselves, establish clinical diagnoses. We have clarified this at first use in the Methods: the cut-offs now explicitly identify participants who *screened positive* for clinically significant anxiety symptoms (GAD-7 ≥ 10) and for panic disorder (PADIS ≥ 4), and we state that the terms “anxiety disorder” and “panic disorder” are used thereafter for brevity to denote screen-positive status rather than confirmed diagnoses **(Pages: 10-11; Lines: 180-193)**. A corresponding sentence has been added to the limitations **(Page: 31; Lines: 515-517)**. We respectfully retain the established terms because they are widely used in this way in the epidemiological literature and because no gold-standard diagnostic measure exists for these conditions; the added clarifications ensure the screening basis is unambiguous to readers*.*

***Annotation 2 (L95, “mimics”):*** *Emotional exertion can precipitates angina as well as anxiety and panic attacks. So anxiety and panic disorders not only mimic but also can co-occur with angina.*

**Response:** We respectfully submit that the sentence in question is accurate as written and already conveys this point: “This diagnostic complexity is compounded by the substantial overlap between cardiac and non-cardiac presentations, particularly in the context of anxiety disorders and panic disorder, which can produce chest pain that closely mimics classical angina both clinically and experientially.” The sentence explicitly acknowledges that anxiety and panic disorders can produce angina-like chest pain. We have therefore retained it unchanged, but we have ensured that the related limitation regarding the specificity of symptom-based classification (the RAQ) makes this overlap explicit.

***Annotation 3 (L144, Methods heading):*** *You should also mention the aims/objectives of the study — primary and secondary objectives.*

**Response:** As noted under General comment G2, the study objectives are stated in the Introduction, which is their appropriate location under STROBE; placing them additionally in the Methods would be inconsistent with that reporting standard. We have expanded the existing closing sentence of the Introduction to distinguish the primary objective (prevalence) from the secondary objectives (characteristics and determinants), which we believe addresses the reviewer’s underlying concern **(Page: 7; Lines: 111-113)**.

***Annotation 4 (L149, “24 and 60”):*** *The study excluded physicians older than 60 years, yet the title and conclusions imply applicability to all physicians in the MENA region. Please clarify the target population and generalizability. It seems that the finding applies to younger physicians only.*

**Response:** We have added a sentence to the limitations noting that eligibility was restricted to physicians aged 24–60 years and that the findings may not generalise beyond this range **(Page: 31; Lines: 522-523)**. We respectfully note that this range already encompasses the large majority of actively practising physicians in the MENA region, and we have therefore not repeated the age range at every mention, which we felt would burden the text without adding clarity.

***Annotation 5 (L162, questionnaire):*** *It would be good if you put the self-made tools in one of the supplementary materials.*

**Response:** We have compiled the author-developed items — with their exact question wording — into a one-page Supporting Information file and reference it in the Methods (Data collection tools). The validated instruments embedded in the survey (RAQ, GAD-7, PADIS) are cited to their original published sources.

***Annotation 6 (L173–174, “social history”):*** *Please recheck whether these variables are appropriately categorized under “social history.” In my view, nicotine use, alcohol consumption, recreational drug use, and caffeinated drink consumption would be better placed under “personal history”. Are you documenting current use or lifetime use? Any cut-off amount or frequency used while documenting substance use?*

**Response:** We thank the reviewer. On terminology, we have relabelled these variables as "personal history" as suggested. On the nature of the measures: the items on alcohol, recreational drug, and caffeinated-drink consumption asked whether participants used each substance (e.g., "Do you drink alcohol?") without specifying a reference period, and were analysed as reported (yes/no); we therefore do not characterise them as strictly current or lifetime use. The nicotine item explicitly distinguished current from former use through its response options (e.g., current tobacco use, current e-cigarette use, previous use). On thresholds: no cut-off amount was applied to define use for any substance; quantity/frequency information (years of use and average cigarettes per day) was collected for nicotine products only.

***Annotation 7 (L177, “psychiatric comorbidities”):*** *Is this also self-reported?*

**Response:** Yes. Psychiatric comorbidities, like physical comorbidities, were self-reported. We have stated this explicitly in the Methods **(Page: 9; Line: 160)** and reinforced it in the limitations (self-report of exposures).

***Annotation 8 (L221, “three chest pain categories”):*** *Which are three chest pain groups? you have only 2 chest pain groups-definite and possible. One group is without chest pain.*

**Response:** We thank the reviewer and have revised the wording to make clear that the chest-pain outcome is a single three-level variable rather than “three chest-pain categories.” The revised sentence reads **(Pages: 11-12; Lines: 208-210)**: “Between-group differences across the three categories of the chest-pain outcome — definitive anginal pain, possible anginal pain, and no chest pain — were examined using…” This makes explicit that the outcome comprises two chest-pain groups (definitive and possible) and a no-chest-pain reference group.

***Annotation 9 (L270, Results):*** *I think the primary objective is to find the prevalence of chest pain. it is good to put that finding first. then it can be presented as 'possible' and 'definite' angina which can be secondary objective.*

**Response:** We thank the reviewer for this helpful suggestion. We agree that the overall prevalence of chest pain is the primary outcome, and that its subdivision into possible and definitive anginal pain represents a secondary, more granular finding. We have accordingly revised the opening of the Results so that the overall prevalence is presented first, followed by the subtype distribution. The revised passage reads **(Page: 14; Lines: 254-257)**: "Overall, 3,631 (58.3%) reported having experienced chest pain, while 2,593 (41.7%) had never experienced chest pain. Among those reporting chest pain, 795 (12.8% of the total sample) met criteria for definitive anginal pain and 2,836 (45.6%) for possible anginal pain."

***Annotation 10 (L282, “mental health and sleep”):*** *Mental health, sleep, anxiety, panic disorder are overlapping constructs. How are you defining mental health here. That should be mentioned in methodology.*

**Response:** We have replaced the umbrella phrase “mental health and sleep” with “psychological and sleep-related factors” at this point in the Results **(Page: 14; Line: 265)**. Each construct is defined operationally in the Methods—screen-positive anxiety symptoms (GAD-7 ≥ 10), screen-positive panic disorder (PADIS ≥ 4), self-reported subjective sleep deprivation, and self-reported psychiatric comorbidity—and is reported separately with its own figures in the paragraph. We note that their conceptual overlap is acknowledged in the Discussion and is addressed by our sensitivity analysis additionally adjusting for psychiatric comorbidity.

***Annotation 12 (L299, Chest pain characteristics):*** *This analysis has not been described in the Methods section. Please include the corresponding methodological details there so the results can be properly interpreted.*

**Response:** We thank the reviewer. The chest-pain characteristics presented in Table 2 (pain site, frequency, triggers, and quality descriptors) are derived directly from the items of the Rose Angina Questionnaire, which is described in the Methods (RAQ subsection); these are reported as descriptive frequencies and required no additional analytical method beyond those already specified.

# Responses to Reviewer #2

**Response:** We are grateful to Reviewer #2 for the positive assessment and for two helpful points, both of which we have addressed.

## 1. Data Availability Statement

***Reviewer 2:*** *The Data Availability Statement does not fully comply with PLOS ONE requirements. Raw data underlying summary statistics, including individual-level responses, should be made publicly available, or restrictions should be explicitly justified.*

**Response:** We thank the reviewer. We have prepared a de-identified minimal dataset and provide it as Supporting Information. Directly and indirectly identifying variables—including exact country of residence, nationality, medical specialty, marital status, academic degree, years of experience, and exact age—were removed because, in combination with the sensitive health and behavioural variables retained (such as psychiatric comorbidity and recreational drug use), they could permit re-identification of individual physician participants, consistent with the PLOS provision that indirect identifiers may be inappropriate to share. The shared dataset reproduces the reported prevalence estimates, the GAD-7 and PADIS classifications, and the chest-pain characteristics, and contains all variables entered into the regression models except age, which was withheld on privacy grounds; the complete adjusted model specifications, including the age-adjusted estimates, are reported in Tables 3 and 4. The revised statement **(Pages: 34-35; Lines: 585-593)** reads:

*“The minimal dataset underlying the reported analyses is provided as Supporting Information. To protect participant privacy, directly and indirectly identifying variables—including exact country of residence, nationality, medical specialty, marital status, academic degree, years of experience, and exact age—were removed, as these fields, particularly in combination with the sensitive health and behavioural variables retained (e.g., psychiatric comorbidity and recreational drug use), could permit re-identification of individual physician participants. The retained variables are sufficient to reproduce the reported prevalence estimates, symptom characteristics, and the direction and significance of the reported associations; full model specifications, including age-adjusted estimates, are reported in Tables 3 and 4.”*

## 2. Causal language in the Conclusion

***Reviewer 2:*** *In the Conclusion, “are major drivers alongside traditional cardiovascular risk factors” may imply causation. Consider “were strongly associated with chest pain alongside traditional cardiovascular risk factors.”*

**Response:** We agree and have adopted the reviewer's suggested wording. The revised statement **(Page: 31; Lines: 527-530)** reads: “Chest pain was highly prevalent among physicians in this large MENA-region cohort, with the pattern of determinants suggesting that psychological, occupational, and behavioral factors — particularly panic disorder, anxiety, sleep deprivation, obesity, and nicotine product use — were strongly associated with chest pain alongside traditional cardiovascular risk factors. ”

# Closing

We believe the revised manuscript is substantially strengthened by these changes and hope it is now suitable for publication in PLOS ONE. We thank the editor and reviewers again for their time and constructive input.

Sincerely,

Weam Aldiban, on behalf of all authors
