# Supplementary material for: Prevalence, characteristics, and determinants of chest pain among physicians in the Middle East and North Africa: A multinational cross-sectional study
Source: PLoS One. 2026 Aug 31;21(8):e0355946. doi: 10.1371/journal.pone.0355946 (PMC13529011; doi:10.1371/journal.pone.0355946)
Supplement: Supplementary file 9 [file pone.0355946.s011.docx]

**Response to Reviewers**

**Manuscript PONE-D-26-19587R1**

*Prevalence, characteristics, and determinants of chest pain among physicians in the Middle East and North Africa: A multinational cross-sectional study*

Dear Dr. Shrestha and Reviewers,

We thank the Academic Editor and both reviewers for their continued time and for the positive assessment of our revised manuscript. We are grateful that both reviewers considered our previous responses satisfactory. We have addressed the two remaining points raised by Reviewer #1 and the journal requirement regarding the reference list. In addition to the points raised by the reviewers, we have made the following changes, which we bring to the editor's attention for transparency.

Figure 1 was regenerated to comply with PLOS ONE technical specifications for figures. In doing so, we also improved its readability: specialties are now ordered by descending prevalence of definitive anginal pain, percentage labels are displayed within the bars, and several specialty labels were standardised for consistency with the text. The chi-squared statistic previously displayed within the image has been moved to the figure caption, in line with PLOS guidance that figure files should not contain caption text. A formal caption has been added to the manuscript. The underlying data and all reported values are unchanged.

We also noted that the sentence describing specialty-specific prevalence had omitted Urology (17.9%), which ranked above Emergency Medicine. This has been corrected so that the text accurately reflects the figure.

Reviewer comments are shown in italics with a coloured rule; our responses follow.

# Response to the Journal Requirements

## 1. Reviewer-recommended citations

***Editor:*** *If the reviewer comments include a recommendation to cite specific previously published works, please review and evaluate these publications to determine whether they are relevant and should be cited. There is no requirement to cite these works unless the editor has indicated otherwise.*

**Response:** The reviewers did not recommend any specific previously published works for citation. No citations were added on this basis.

## 2. Reference list and retracted articles

***Editor:*** *Please review your reference list to ensure that it is complete and correct. If you have cited papers that have been retracted, please include the rationale for doing so in the manuscript text, or remove these references and replace them with relevant current references. Any changes to the reference list should be mentioned in the rebuttal letter that accompanies your revised manuscript. If you need to cite a retracted article, indicate the article’s retracted status in the References list and also include a citation and full reference for the retraction notice.*

**Response:** We have carefully reviewed the complete reference list and verified each citation against the original published source and against the Retraction Watch Database and Crossref retraction notices. No retracted articles were identified among the cited references, and no changes to the reference list were required. All references were confirmed to be complete and correctly formatted.

# Responses to Reviewer #1

**Response:** We thank Reviewer #1 for the positive assessment of the revised manuscript and for these two final, constructive suggestions, both of which we have implemented.

## 1. Definition of the abbreviation ENDS

***Reviewer 1:*** *The abbreviation ENDS was used on multiple occasions without prior definition. It is standard practice to provide the full term alongside the abbreviation when you use it for the first time in the text.*

**Response:** We thank the reviewer for identifying this oversight and apologise for the inconsistency. We have now defined the abbreviation in full at its first occurrence in both the Abstract and the main text, as the Abstract is read independently of the article. In the Abstract, the sentence now reads: “…while electronic nicotine delivery system (ENDS) use was significant only for possible anginal pain.” In the main text, the term is defined at first mention in the Results. We have additionally reviewed the entire manuscript, including all tables and figure legends, to ensure the abbreviation is used consistently and in capitalised form throughout (e.g., “Exclusive ENDS use” in Table 1, previously rendered in lower case).

## 2. Implications of the study findings

***Reviewer 1:*** *Also, you can add the implication of the finding of your study in the manuscript.*

**Response:** We agree that the implications of our findings warranted greater prominence. We have introduced a dedicated subsection headed “Implications of the findings” in the Discussion, which now presents the practical implications for occupational health policy and clinical practice explicitly, and we have expanded it to address the research implications of the study. The added text reads “Beyond immediate practice, these findings identify clear priorities for future research. Prospective studies incorporating objective cardiac endpoints — including electrocardiography, biomarkers, and imaging — are needed to determine whether the symptom burden observed here translates into measurable cardiovascular risk over time. Intervention studies are also required to establish whether targeted improvements in sleep and psychological health reduce chest pain burden in this workforce, and whether such interventions are feasible within the constraints of clinical training and practice. Finally, the observed differences by country income level highlight the need for health-system-level research examining how staffing, workload, and access to occupational health services shape symptom burden across diverse healthcare settings.”

# Response to Reviewer #2

***Reviewer 2:*** *Thank you for addressing the issues raised in peer review. I believe this is a great article - While treating patients, physicians underestimate the impact of their profession, lifestyle and mental health on their personal overall health and large cross-sectional studies like these generate new questions to be answered and will help guide research in the right direction.*

**Response:** We sincerely thank Reviewer #2 for this generous assessment and for the constructive engagement throughout both rounds of review. We share the reviewer’s view that the value of this work lies partly in the questions it raises for future investigation, and we have reflected this explicitly in the new “Implications of the findings” subsection described above.

# Closing

We are grateful to the Academic Editor and both reviewers for their careful and constructive input across both rounds of review, which has substantially improved the manuscript. We hope the revised version is now suitable for publication in PLOS ONE.

Sincerely,

Weam Aldiban, on behalf of all authors
